# Stem cell microencapsulation maintains stemness in inflammatory microenvironment

**DOI:** 10.1038/s41368-022-00198-w

**Published:** 2022-10-10

**Authors:** Yajun Zhao, Yilin Shi, Huiqi Yang, Mengmeng Liu, Lanbo Shen, Shengben Zhang, Yue Liu, Jie Zhu, Jing Lan, Jianhua Li, Shaohua Ge

**Affiliations:** 1 Department of Implantology & Periodontology, School and Hospital of Stomatology, Cheeloo College of Medicine, Shandong University & Shandong Key Laboratory of Oral Tissue Regeneration & Shandong Engineering Laboratory for Dental Materials and Oral Tissue Regeneration & Shandong Provincial Clinical Research Center for Oral Diseases, Jinan, China; 2 Key Laboratory of Shaanxi Province for Craniofacial Precision Medicine Research, College of Stomatology, Xi’an Jiaotong University, Xi’an, China; 3 Airong Plastic Surgery Hospital, Jinan, China

**Keywords:** Mesenchymal stem cells, Nanobiotechnology

## Abstract

Maintaining the stemness of the transplanted stem cell spheroids in an inflammatory microenvironment is challenging but important in regenerative medicine. Direct delivery of stem cells to repair periodontal defects may yield suboptimal effects due to the complexity of the periodontal inflammatory environment. Herein, stem cell spheroid is encapsulated by interfacial assembly of metal-phenolic network (MPN) nanofilm to form a stem cell microsphere capsule. Specifically, periodontal ligament stem cells (PDLSCs) spheroid was coated with Fe^III^/tannic acid coordination network to obtain spheroid@[Fe^III^-TA] microcapsules. The formed biodegradable MPN biointerface acted as a cytoprotective barrier and exhibited antioxidative, antibacterial and anti-inflammatory activities, effectively remodeling the inflammatory microenvironment and maintaining the stemness of PDLSCs. The stem cell microencapsulation proposed in this study can be applied to multiple stem cells with various functional metal ion/polyphenol coordination, providing a simple yet efficient delivery strategy for stem cell stemness maintenance in an inflammatory environment toward a better therapeutic outcome.

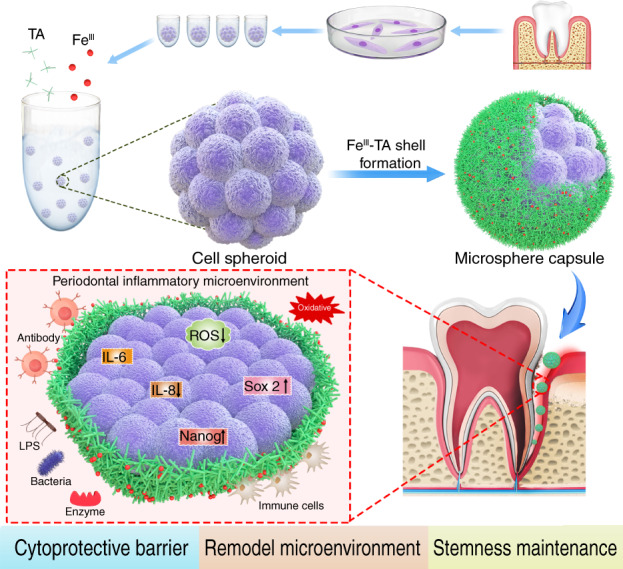

## Introduction

Tissue repair following physical injury or microbial infection is a complex and tightly regulated process involving a dynamic inflammatory microenvironment, which consists of various inflammatory molecules such as cytokines, proteases, and chemokines in the early stage, and shifts to an anti-inflammatory microenvironment to restore endogenous tissue repair and regeneration in the later stage.^1^ Under normal physiological conditions, the inflammatory response is classically recognized as an essential step in maintaining tissue homeostasis under a variety of deleterious conditions. However, persistent chronic inflammation and infection always lead to the increased production of inflammatory chemokines and cytokines, which could impair the tissue healing process and further worsen the chronic disease.^2^

Stem cells, especially stem cell spheres, hold great promise for the repair and reconstruction of the damaged tissues and organs and they have been utilized for the treatment of chronic inflammation.^3,4^ Currently, clinical applications of stem cell therapy are still limited, as the surrounding environment niche greatly affects the regulation of cell development and function under both physiological and pathological conditions.^5^ Simple transplantation of stem cells into the harsh microenvironment of an inflammatory site could directly expose cells to the accumulated oxidative stress, pathogenic microbes, immune cells and proteolytic enzymes that may lead to poor cell viability, loss of stemness or impaired engraftment.^6–8^ For instance, periodontitis is a globally recognized classic infection-driven chronic inflammatory disease, characteristically causing destruction and absorption of alveolar bone.^7,9^ Periodontal ligament stem cells (PDLSCs) have been demonstrated to generate cementum/ periodontal ligament-like tissue in vivo and are recognized as potential seed cells for periodontal tissue regeneration.^10^ However, bacteria (e.g. *Porphyromonas gingivalis*, *P. gingivalis*) products, immune cells and inflammatory mediators in periodontal pockets, such as lipopolysaccharide (LPS), reactive oxygen species (ROS) and hydrolytic enzymes, collectively hinder the therapeutic capacity of the transplanted PDLSCs.^11^ Therefore, there is a dire need to develop an optimized stem cell delivery strategy to maximize their therapeutic capacities.

Cell encapsulation is an emerging technique that envelopes live cells in a semipermeable membrane, showing great potential for stem cell transplantation.^12^ One typical enveloping membrane is the use of hydrogel, whose thickness is usually ranging from micrometer to millimeter scale that is not conducive to the exchange of nutrients, oxygen and metabolic waste, and unfavorable for rapid response to changes in the host environment.^13,14^ Moreover, most encapsulation materials usually act as physical barriers and fail to actively manipulate the surrounding environmental niche,^15,16^ which potentially limits their clinical applications under inflammatory conditions.

In recent years, metal-phenolic network (MPN), a supramolecular non-covalent coordination nanofilm with semipermeable features, has attracted great attention and is considered to be promising for the construction of bioactive interfaces.^17,18^ MPN composed of different metal ions and phenolic ligands exhibits excellent antioxidative, anti-inflammatory and antibacterial functions.^19–22^ To date, due to its excellent biocompatibility and universal adhesion, application of MPN nanofilm in coating single bacteria, yeast, and mammalian cell has been realized.^20,23,24^ Compared with individual cell, stem cell microspheres or aggregates have superior therapeutic capacities,^25–27^ nevertheless, nanocoating for stem cell microsphere using MPN as bioactive interface is scarcely studied.

In this study, we developed a stem cell microsphere enveloped in an MPN capsule as delivery vehicle for PDLSCs. ROS, *P. gingivalis* and LPS, etc. in periodontitis were used as inflammatory factor models to study the function of stem cell microsphere capsule. Then, we investigated whether the MPN shell could effectively remodel the inflammatory microenvironment in an antioxidative, antibacterial and anti-inflammatory manner and maintain the stemness of PDLSCs. To further study the plausible mechanism of MPN encapsulation on stem cells, an RNA-sequencing analysis was performed to reveal the relationship between genetic alteration and stemness maintenance after MPN encapsulation.

## Results

### Fabrication and characterization of stem cell microsphere capsule

**Fig. 1:**
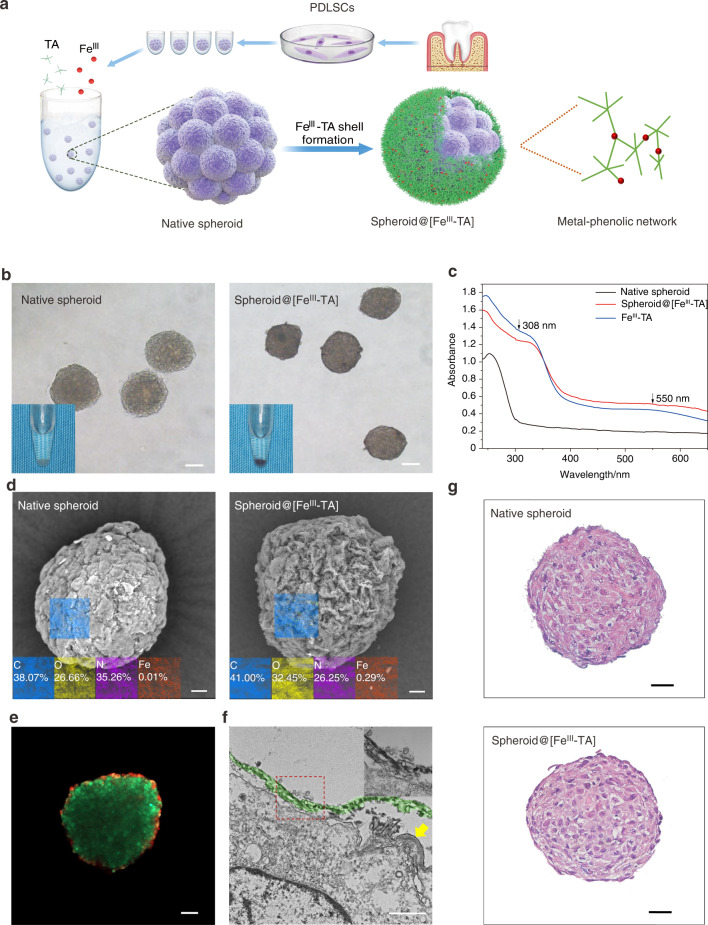
Fabrication and characterization of a stem cell microsphere capsule. **a** Schematic illustration for nanocoating formation of Fe^III^ and TA on the surface of a PDLSCs spheroid. **b** Phase contrast microscope images of native spheroid and spheroid@[Fe^III^-TA]. Inset, photograph of native spheroid and spheroid@[Fe^III^-TA]. Scale bar = 50 μm. **c** UV–Vis absorption spectra of native spheroid, spheroid@[Fe^III^-TA], and Fe^III^-TA. **d** SEM images and EDS elemental analysis of native spheroid and spheroid@[Fe^III^-TA]. Scale bar = 10 μm. **e** CLSM image of spheroid@[Fe^III^-TA] after treated with RhB. Scale bar = 50 μm. **f** TEM micrograph of spheroid@[Fe^III^-TA]_._ The red dashed line indicated the area of interested magnified images. The yellow arrow indicated the cell membrane. Scale bar = 1 μm. **g** H&E staining of native spheroid and spheroid@[Fe^III^-TA]. Scale bar = 20 μm

As illustrated in Fig. 1a, a PDLSC spheroid was enveloped with Ferric ion (Fe^III^)-tannic acid (TA) coordinated supramolecular network. The Fe^III^-TA nanocoating was formed on the PDLSC spheroid by simply adding TA and FeCl_3_·6H_2_O sequentially to the suspension of cell spheroids that were previously formed in a non-adhesive agarose hydrogels mold. The successful self-assembly of the Fe^III^-TA MPN shell on the cell spheroids (hereafter denoted spheroid@[Fe^III^-TA]) was indicated by a noticeable color change. Compared with the white native spheroids, the coated ones appeared as purple black pellets (Fig. 1b). The color change caused distinct changes in the absorption peaks of the UV–Vis absorption spectrum. As shown in Fig. 1c, the broad peak around 550 nm corresponds to the typical coordinated TA to Fe^III^-charge transfer band in Fe^III^-TA coordination compounds^28,29^ and an accompanying shoulder appears at ~308 nm suggested the conjugation between Fe^III^ and the deprotonated form of TA. The presence of Fe element in the nanocoating was verified by energy dispersive spectroscopy (EDS) equipped scanning electron microscopy (SEM) (Fig. 1d), the relatively low loading of Fe (~0.29% atomic percent) could avoid bringing cytotoxicity to the coated cell spheroids. Fourier transform infrared spectroscopy (FT-IR) was used to confirm the typical functional groups of the nanocoating. The absorption peak centered at 1 640 cm^−1^ and 1 710 cm^−1^ was assigned to the C=C distortion vibration from benzene rings and the ester groups of TA, respectively^30,31^ (Fig. S1).

The core–shell structure was visualized by labeling the Fe^III^-TA shell with Rhodamine B (RhB) dye molecule during MPN assembly,^32^ and staining the live cells with calcein-AM probe after capsule formation. As shown in Fig. 1e, an obvious red-fluorescent ring on the surface of viable cell spheroid was observed by the confocal laser-scanning microscopy (CLSM) images. The micrograph obtained from transmission electron microscopy (TEM) clearly showed a uniform Fe^III^-TA shell with an average thickness about 40 nm on the surface of cell spheroid (Fig. 1f), which was consistent with the previous report.^19^ From the hematoxylin and eosin (H&E) staining images of native spheroid and spheroid@[Fe^III^-TA], the nanocoated cell spheroid maintained their original shape with little difference in internal morphology (Fig. 1g).

### Molecular gating of the Fe^III^-TA shell based on permeability

**Fig. 2:**
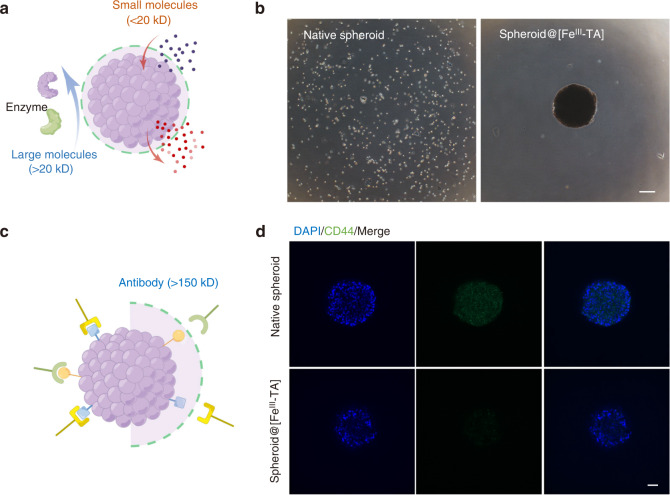
Molecular gating of the Fe^III^-TA shell based on permeability. **a** A schematic image about the selective permeability of the Fe^III^-TA shell. **b** Phase contrast microscope image of native spheroid and spheroid@[Fe^III^-TA] after incubation with trypsin solution for 30 min. Scale bar = 100 μm. **c** A schematic image about the cytoprotective ability of the Fe^III^-TA shell in antibody immunoreaction. **d** CLSM images of native spheroid and spheroid@[Fe^III^-TA] after immunostaining. Green, anti-CD44. Blue, DAPI. Scale bar = 50 μm

In chronic inflammatory periodontal diseases, uncontrolled release of host- and bacteria-derived proteases can lead to the degradation of extracellular matrix, which not only causes self-digestion and tissue destruction,^33^ but also impairs the viability of transplanted stem cells. In addition, a harsh in vivo environment, including immune cells and antibodies, can also lead to immune rejection or cellular damage. Therefore, protecting the encapsulated cell spheroids against the attacks from the surrounding protease and antibody is a treatment goal to modulate the periodontal inflammatory microenvironment. Notably, the Fe^III^-TA shell is selectively permeable (Fig. 2a). Small molecules with low *M*_w_ (<20 kD) diffuse freely across the shell, while large molecules with high M_w_ (i.e., 2 000 kD) are almost impermeable.^34^ To verify the cytoprotective role of the encapsulation layer against protease attack, trypsin (24 kD) was utilized as a model of proteolytic enzymes. After incubation with trypsin solution for 30 min, native spheroids were digested into single cells while spheroid@[Fe^III^-TA] remained intact (Fig. 2b).

Furthermore, anti-CD44 (>150 kD) was used to detect the blocking effect of the Fe^III^-TA shell (Fig. 2c) on antibodies. CD44 antigens are cell-surface glycoprotein widely expressed on mesenchymal stem cells and participate in various cellular functions such as cellular adhesion and migration.^35^ As shown in Fig. 2d, native spheroids were stained with green fluorescence while little green fluorescence was seen inside the spheroid@[Fe^III^-TA], which indicated little penetration of anti-CD44 across the Fe^III^-TA encapsulated layer. These data suggested that the Fe^III^-TA shell could protect stem cells from enzymatic digestion and antibody immunoreaction by acting as a molecular gating barrier based on its selective permeability.

### ROS scavenging activity of the Fe^III^-TA shell

**Fig. 3:**
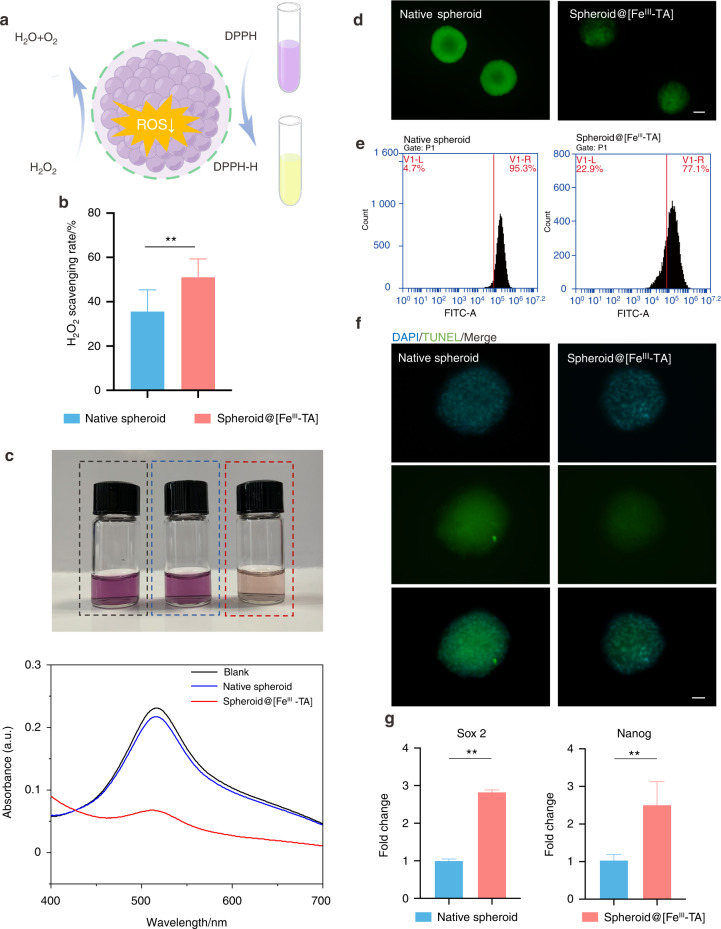
In vitro antioxidative activity of the Fe^III^-TA shell. **a** A schematic image about the antioxidative activity of the Fe^III^-TA shell. **b** H_2_O_2_ scavenging rate after a 2 h incubation with cell spheroids. **c** UV–Vis absorption spectra of the catalyzed oxidation of DPPH on different groups. The corresponding digital photos of each group were shown below. **d**, **e** Intracellular ROS level of native spheroid and spheroid@[Fe^III^-TA] determined by fluorescence images and flow cytometry analysis. Scale bar = 100 μm. **f** Apoptosis detection by TUNEL staining of cell spheroids after 2 h incubation with H_2_O_2_. Scale bar = 50 μm. **g** qRT-PCR measurements of *Sox2* and *Nanog* expression in native spheroid and spheroid@[Fe^III^-TA] after 24 h incubation with H_2_O_2_. **P < 0.01

Increased levels of local and systemic oxidative stress promote the inflammatory microenvironment of periodontitis and lead to impaired antioxidant capacity.^7^ ROS is produced as normal aerobic metabolism byproducts and can be effectively balanced by antioxidants under physiological conditions. However, when inflammation occurs, ROS increases dramatically and cannot be neutralized by the antioxidants, leading to oxidative stress and tissue impairment.^36^ Thus, a smart strategy of stem cell transplantation should include a dynamic scavenge in response to excessive ROS (Fig. 3a). Hydrogen peroxide (H_2_O_2_) was widely used in cellular models to induce oxidative stress as an exogenous ROS. The H_2_O_2_ scavenging capacity of the Fe^III^-TA shell was then tested. As shown in Fig. 3b, compared with native spheroids, the spheroid@[Fe^III^-TA] exhibited stronger H_2_O_2_ scavenging capacity. In vivo, catalase (CAT) catalyzed the decomposition of H_2_O_2_. Similarly, the Fe^III^-TA shell possessed favorable CAT-like activity. Total ROS scavenging activity was measured by 1,1-diphenyl-2-picrylhydrazyl (DPPH) assay. As shown in Fig. 3c, the color of DPPH solution incubated with spheroid@[Fe^III^-TA] was lighter than that incubated with native spheroid. A sharp absorption peak of DPPH was observed at 570 nm in the blank and native spheroid group. Therefore, the ROS scavenging activity of spheroid@[Fe^III^-TA] was significantly enhanced compared with native spheroids.

To verify the effect of the Fe^III^-TA shell on H_2_O_2_-induced (300 μmol·L^−1^, mimicking pathological oxidative stress) intracellular oxidative stress status, ROS production in cell spheroids was assessed. As shown in Fig. 3d, ROS level elevated in native spheroid by H_2_O_2_ treatment compared with spheroid@[Fe^III^-TA]. Flow cytometry analysis also confirmed the result that spheroid@[Fe^III^-TA] had lower ratios of intracellular ROS compared with native spheroid (Fig. 3e), indicating that the Fe^III^-TA shell attenuated H_2_O_2_-induced oxidative stress and exerted antioxidant effects on PDLSC spheroids under simulated oxidative stress status. To investigate whether the Fe^III^-TA shell decreased H_2_O_2_-induced cell apoptosis, terminal deoxynucleotidyl transferase (TdT) dUTP nick end labeling (TUNEL) assay was performed to detect apoptotic cells. The results indicated that more FITC-labeled TUNEL-positive cells could be observed in native spheroid than in spheroid@[Fe^III^-TA] upon H_2_O_2_ stimulation (Fig. 3f). The relative mRNA expression of pluripotent markers *Sox2* and *Nanog* was also examined.^37^ As shown in Fig. 3g, quantitative RT-PCR analysis showed that after incubation with H_2_O_2_ for 24 h, *Sox2* and *Nanog* mRNA expression levels were significantly downregulated in native spheroid compared with spheroid@[Fe^III^-TA]. Considering that TA is a naturally occurring antioxidant polyphenol, the Fe^III^-TA shell with TA as the main component also possessed antioxidant activity. H_2_O_2_ stimulation induced stemness loss in native spheroid, whereas the Fe^III^-TA shell attenuated the H_2_O_2_ damage. These data indicated that the antioxidative activity of the Fe^III^-TA shell could function in a microenvironment-responsive manner and effectively maintained cell stemness in an oxidative stress microenvironment.

### Antibacterial and anti-inflammatory activity of the Fe^III^-TA shell

**Fig. 4:**
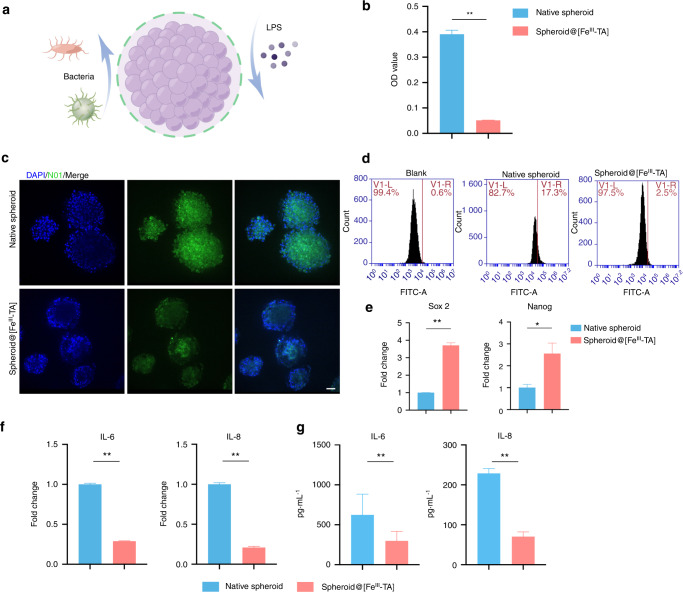
In vitro antibacterial and anti-inflammatory activity of the Fe^III^-TA shell. **a** A schematic image about the antibacterial activity of the Fe^III^-TA shell. **b** Optical density values of *P. gingivalis* after incubation with native spheroid and spheroid@[Fe^III^-TA] for 24 h. **c**, **d** Intracellular *P. gingivalis* of native spheroid and spheroid@[Fe^III^-TA] determined by fluorescence images and flow cytometry analysis. Scale bar = 50 μm. **e** qRT-PCR measurements for mRNA expression level of *Sox2* and *Nanog* in native spheroid and spheroid@[Fe^III^-TA] after 24 h incubation with *P. gingivalis*. **f**, **g** qRT-PCR and ELISA measurements for pro-inflammatory genes (*IL-6* and *IL-8*) in native spheroid and spheroid@[Fe^III^-TA] after 6 h incubation with LPS. *P < 0.05, **P < 0.01

Periodontitis is an inflammatory disease caused by subgingival plaque biofilm infection and followed by a host immune response.^38^ Among periodontal pathogens, *P. gingivalis* is a key candidate with many potential virulence factors associated with the onset of periodontitis.^39^ Studies have confirmed that *P. gingivalis* can locally invade periodontal tissues^40,41^ and subsequently affect cellular functions, such as differentiation, migration and apoptosis.^6,42,43^ In the inflammatory microenvironment of periodontitis, the activity and function of exogenous stem cells are also threatened by bacterial invasion. Given the efficient antimicrobial activity of TA based on a previous study,^44^ we further explored whether the Fe^III^-TA nanocoating strategy would combat *P. gingivalis* (Fig. 4a). First, 6-well plates were coated with Fe^III^-TA at the same concentration and layers as the spheroid@[Fe^III^-TA] and then *P. gingivalis* was inoculated into the wells. As shown in Fig. S2a, after incubation for 24 h, the OD_600_ value of the Fe^III^-TA nanocoated group was statistically lower than the control group, indicating that the Fe^III^-TA nanocoating could suppress the proliferation of *P. gingivalis*. The biofilm formation on the bottom of the plate was detected using bacterial staining kit. Without Fe^III^-TA nanocoating, biofilm formed on the surface of plate was stained green, indicating that the bacteria were alive in good conditions. Noticeably, with Fe^III^-TA nanocoating, few bacteria stained in green were visible on the bottom of plate, which also demonstrated the antibacterial activity of the Fe^III^-TA nanocoating (Fig. S2b).

Then we investigated whether the Fe^III^-TA shell could attenuate the damage of *P. gingivalis*. Cell spheroids were infected with *P. gingivalis* at a multiplicity of infection (MOI) of 1 000. After 24 h, the OD_600_ value of *P. gingivalis* co-cultured with spheroid@[Fe^III^-TA] was statistically lower than with native spheroid (Fig. 4b). Fluorescence images showed that the number of pre-labeled green fluorescent bacteria entering native spheroid was significantly higher than that of spheroid@[Fe^III^-TA] (Fig. 4c), which was also confirmed by flow cytometry analysis (Fig. 4d). Furtherly, we examined the expression of stemness genes in cell spheroids after 24 h incubation. As shown in Fig. 4e, quantitative RT-PCR analysis indicated that the expression of *Sox2* and *Nanog* was significantly downregulated in native spheroid compared with spheroid@[Fe^III^-TA]. Bacterial infection resulted in reduced stemness in native spheroid, whereas the antibacterial activity of the Fe^III^-TA shell attenuated the damage from *P. gingivalis*. These data demonstrated that the antibacterial Fe^III^-TA shell could help maintain the stemness of cell spheroids when invaded by bacteria.

The virulence factors of *P. gingivalis*, such as LPS, not only directly impair the periodontal supporting tissues, but also cause secondary tissue damage by generating an inflammatory response.^45,46^ LPS and its lipid A moiety stimulate cells by Toll-like receptor 4 (TLR4) and can promote the expression levels of pro-inflammatory *interleukin (IL)-6* and *IL-8* in PDLSCs.^47^ Intact LPS is a macromolecule with a molecular weight of 10–20 kD that forms aggregates of varying sizes and structures in aqueous media.^48^ Considering the molecular gating role based on selective permeability, we hypothesized that the stem cell microsphere capsule could partly protect the inside cells from LPS stimulation. Furthermore, TA has been shown to suppress LPS-induced macrophage polarization towards M1 by inhibiting the binding between LPS and TLR4.^49^ In order to verify the anti-inflammatory activity of the Fe^III^-TA shell, we then explored the effect of LPS on the expression of inflammatory factors in native spheroid and spheroid@[Fe^III^-TA]. After stimulation with LPS for 6 h, both the mRNA and protein expression of *IL-6* and *IL-8* was significantly lower in spheroid@[Fe^III^-TA] compared with native spheroid (Fig. 4f, g), indicating that in addition to stemness maintenance, the Fe^III^-TA encapsulation also regulated the immunomodulatory function of PDLSC spheroids.

### Maintenance of viability and stemness of the spheroid@[Fe^III^-TA]

**Fig. 5:**
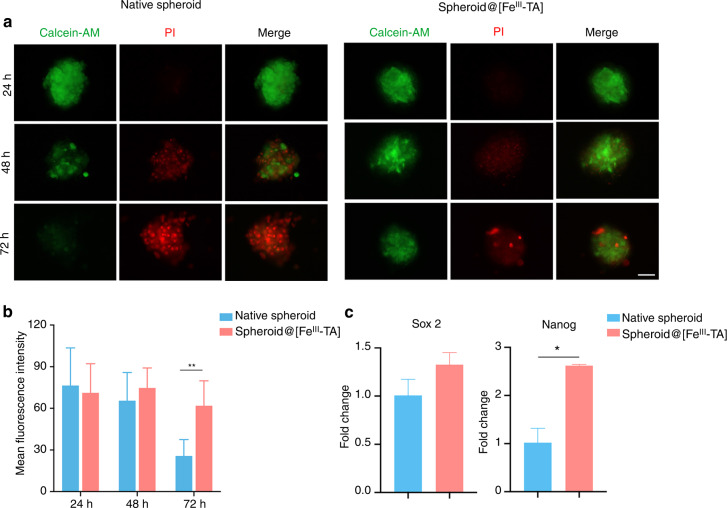
Maintenance of viability and stemness of spheroid@[Fe^III^-TA]. **a** Live/Dead cell viability assay of native spheroid and spheroid@[Fe^III^-TA] after coating for 24, 48, 72 h. Green, live; red, dead. Scale bar = 50 μm. **b** Mean fluorescence intensity analysis of calcein-AM. **c** qRT-PCR measurements for mRNA expression level of *Sox2* and *Nanog* in native spheroid and spheroid@[Fe^III^-TA] after coating for 24 h. *P < 0.05

The loss of stem cell viability and stemness is a major challenge during the in vitro culture of stem cell spheroids and after cell implantation in vivo.^50,51^ The antioxidative and antibacterial activities of the Fe^III^-TA shell has been shown to effectively maintain the stemness of inner cell spheroids under noxious external stimuli. However, it is unclear whether the Fe^III^-TA shell itself can maintain the viability or stemness of the PDLSC spheroids during in vitro culture. After encapsulation, cell viability was investigated at 24, 48 and 72 h with live/dead staining. Cellular viability decreased both in native spheroid and spheroid@[Fe^III^-TA], but there were more live cells in spheroid@[Fe^III^-TA] than native spheroids after 72 h culture (Fig. 5a, b), which indicated that encapsulation of the Fe^III^-TA shell was conductive to the maintenance of cell viability. The expression of stemness genes in the cell spheroids was also examined. As shown in Fig. 5c, the expression level of *Sox2* in spheroid@[Fe^III^-TA] was comparable with that in native spheroids and spheroid@[Fe^III^-TA] exhibited higher mRNA levels of *Nanog* than native spheroids. These data suggested that the expression of stemness genes in PDLSC spheroids was maintained after Fe^III^-TA shell encapsulation. The underlying mechanism might be as follows. With the prolonged in vitro culture, nutrient-deficient condition induced high level of ROS formation,^52^ which further damaged the viability and stemness of cell spheroids. However, the antioxidant activity of the Fe^III^-TA shell attenuated oxidative stress-induced cell damage, which maintained the viability and stemness in spheroid@[Fe^III^-TA] rather than native spheroid.

### Differentially expressed genes after encapsulation of the Fe^III^-TA shell

**Fig. 6:**
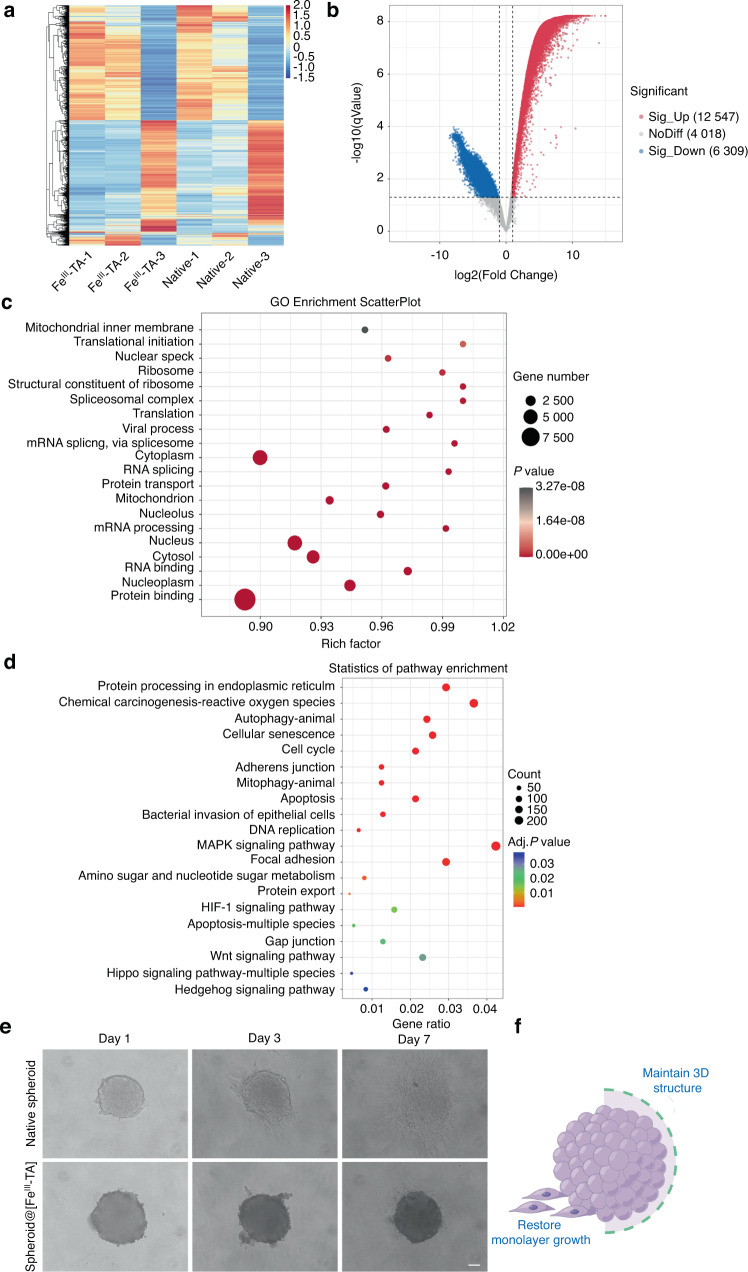
Heatmap, volcano plot, GO, and KEGG functional enrichment results of DEGs upon encapsulation of the Fe^III^-TA shell. **a** Heatmap of the DEGs between native spheroids and spheroid@[Fe^III^-TA]. Red stripes in the figure represented high-expression genes, while blue stripes represented low expression genes. **b** Volcano map of DEGs between native spheroid and spheroid@[Fe^III^-TA]. **c** Scatter diagram of GO functional enrichment analysis of DEGs. **d** Scatter diagram of KEGG pathways. **e** Phase contrast microscope images of native spheroid and spheroid@[Fe^III^-TA] after seeding onto tissue culture polystyrene dishes. Scale bar = 50 μm. **f** A schematic image about improved cell-cell contact after MPN encapsulation. DEGs differentially expressed genes, GO Gene Ontology, KEGG Kyoto Encyclopedia of Genes and Genomes

To reveal the gene expression of PDLSCs associated with the Fe^III^-TA encapsulation, a differentially expressed gene (DEG) analysis has been conducted to determine gene expression differences between native spheroid and spheroid@[Fe^III^-TA]. By sequencing, a total of 18 856 DEGs (|fold change| > 2 and *P* < 0.05) were detected between the native spheroid and spheroid@[Fe^III^-TA] cDNA libraries, of which 12 547 genes were upregulated (higher expression in spheroid@[Fe^III^-TA]) and 6 309 genes were downregulated (Fig. 6a, b). This result indicated a great alteration in gene expression with Fe^III^-TA encapsulation. Then, we performed GO term enrichment on gene sets. A total of 11 168 GO terms were annotated in the biological process category, the cellular component category contained 1 656 GO terms, and 3 829 GO terms in the molecular function category (Fig. S3). The 20 most significant GO enrichment with the smallest false discovery rate (FDR) value were selected to make a scatter plot (Fig. 6c). According to the functional enrichment results, the top 5 GO enrichments were primarily associated with protein binding, nucleoplasm, RNA binding, cytosol and nucleus, among which protein and RNA binding might be related to the barrier function of the Fe^III^-TA shell.

Next, a KEGG pathway enrichment analysis was conducted to identify the most significantly enriched pathways for DEGs. The result showed that a total of 143 significant pathways were identified (Table S1). Notably, DEGs-enriched pathways such as mitogen-activated protein kinases (MAPK) signaling pathway, Hippo signaling pathway, Wnt signaling pathway, etc. were related to cell viability and stemness and the upregulated DEGs were associated with several cellular junction pathways, including gap junction and adherent junction that may be attributed to cell encapsulation (Fig. 6d, Table S2). To verify whether the Fe^III^-TA shell could enhance cellular junctions, we observed changes in spheroid morphology after encapsulation in a conventional culture dish (Fig. 6e). Suspended native spheroids exhibited a loss in spherical structure, with cells developing filopodial structures and restoring monolayer growth in a short time, whereas the spheroid@[Fe^III^-TA] maintained the spherical shape without any observable attachment to the bottom of the petri dish. After 3 days in culture, a very noticeable loose structure could be observed on the outer surface of the spheroid@[Fe^III^-TA], while the inner spheroid remained intact and spherical, which was due to the degradation of the outer Fe^III^-TA shell. When the shell was further degraded, spheroid@[Fe^III^-TA] was observed to attach to the bottom of the petri dish, and the cells crawled out from the edge of the spheroid and became more elongated. Improved cell–cell contact can facilitate stemness maintenance in three-dimensional (3D) structures.^53^ The presence of Fe^III^-TA shell maintained the 3D structure of the cell spheroid with tighter inner cell-cell junctions (Fig. 6f), which might explain the maintenance of cell viability and stemness after Fe^III^-TA encapsulation. Furthermore, DEGs enriched in pathways such as chemical carcinogenesis-reactive oxygen species and bacterial invasion of epithelial cells might indicate the antioxidative and antibacterial activities of the Fe^III^-TA shell, which provided a possible explanation for the above-mentioned results on the Fe^III^-TA shell properties.

## Discussion

In order to obtain the desired therapeutic effect in periodontal tissue regeneration, it is necessary to maintain the viability and stemness of the transplanted stem cells. Although there are many strategies to maintain stemness during in vitro expansion, stemness maintenance after implantation still faces some challenges.^54^ Dysregulated inflammation in periodontitis negatively affects wound healing and tissue regeneration processes,^2^ as the cellular microenvironment plays a significant role in regulating the stemness properties of stem cells, and the inflammatory environment provides a pathologic niche for the transplanted cells, leading to stemness loss and undesired therapeutic outcomes. Thus, it is necessary to develop innovative delivery strategies that can not only manipulate the stem cells to ensure survival and stemness maintenance, but also optimize the surrounding inflammatory environment niche to maximize the ability of transplanted cells in tissue regeneration. In this study, we presented a simple and biocompatible stem cell microsphere capsule that could efficiently act as a cytoprotective barrier, remodel the inflammatory microenvironment, and maintain the stemness of PDLSCs.

TA and Fe^III^ were chosen to form the MPN shell because they are recognized as safe by the U.S. Food and Drug Administration.^18^ Furthermore, the Fe^III^-TA shell has been shown to be highly biocompatible and has been applied in coating individual living cells without significant loss of cell viability.^19,23^ Microsphere capsules are formed upon film deposition by mixing TA and Fe^III^ in cell spheroids suspension. For cell encapsulation, it is vital that the cells retain their function and release bioactive substance inside the encapsulation layer while they are also protected from the host immune system.^55,56^ In our study, the Fe^III^-TA shell exhibited a blocking effect on large molecules such as trypsin or antibody. Meanwhile, spheroid@[Fe^III^-TA] could be stained with the fluorescent dyes calcein-AM (994.86 Da) and propidium iodide (668.40 Da) in the live/dead assay, which indicated that small molecules could freely pass through the Fe^III^-TA encapsulation layer. Thus, the MPN shell was selectively permeable and provided a molecular gating barrier for inner cells.

The extracellular microenvironment is vital for maintaining cell viability and function. To date, few cell encapsulation strategies enhance cellular survival and stemness by manipulating the surrounding environmental niche. In this study, the stem cell microsphere capsule could remodel the inflammatory microenvironment for better cell implantation. That is, antioxidative activity of the MPN shell could not only scavenge extracellular oxidative stress, but also reduce the intracellular ROS levels. The MPN shell exhibited antibacterial activity and attenuated the invasion of *P. gingivalis* to inner cells. Inhibition of LPS-induced production of pro-inflammatory factors demonstrated the anti-inflammatory property of the MPN shell. Most importantly, the stem cell microsphere capsule efficiently maintained the viability and stemness of PDLSCs, suggesting that the combination of these properties may potentially improve cell survival and differentiation after transplantation at inflammatory sites.

Another important property of the Fe^III^-TA shell was its degradability, through which stem cells migrate and differentiate in an optimized inflammatory microenvironment to repair and regenerate damaged tissues. The degradation of Fe^III^-TA coordination film is pH-dependent, and low pH will accelerate the disassembly.^10^ In chronic periodontitis, the microenvironment of periodontal pockets is at a neutral pH, so we examined the degradation behavior of the Fe^III^-TA shell in conventional culture medium and observed that the Fe^III^-TA shell could be degraded within a week and cells within the spheroid remain viable after degradation, which ensures that the migrated cells from spheroid@[Fe^III^-TA] play an active role in the subsequent process of tissue regeneration.

The repair process of tissue damage begins with an early inflammatory response and then initiates a regenerative phase. Tissue regeneration cannot be achieved until inflammation subsides, which takes a certain amount of time (usually several days).^57^ In our study, the presence of MPN could not only remodel the inflammatory microenvironment and shorten the inflammatory response time, but more importantly, it could maintain the stemness of the inner cells during the inflammatory stage. Stemness maintenance indicates enhanced differentiation potential.^58^ As the MPN shell degrades over a few days, its stemness maintenance effect diminishes and cell differentiation begins, which is conductive to the later tissue regeneration.

**Fig. 7:**
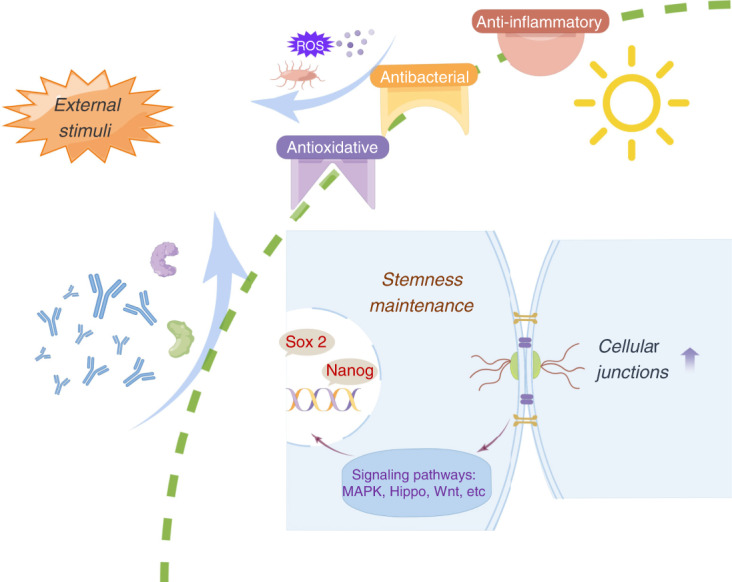
Proposed mechanism of maintaining stem cell microsphere stemness via MPN encapsulation in a periodontal inflammation microenvironment

In conclusion, there were three possible mechanisms that contribute to the stemness maintenance of stem cell microsphere capsule in an inflammatory microenvironment (Fig. 7). First, the supramolecular MPN shell served as a physical molecular gating barrier based on its selective permeability to protect the inner cell spheroid from harmful external stimuli in the inflammatory microenvironment. Second, the antioxidative, antibacterial and anti-inflammatory properties of MPN coating could remodel the microenvironment for better cell implantation. Third, increased cell-cell contact within MPN capsule could promote stemness maintenance within the microsphere.

Due to the complexity of the periodontal inflammatory environment, simply transplanting stem cells to repair specific defects may not achieve the expected regenerative effect. Current researches not only focus on improving the performance of the seed cells, but also consider building and maintaining a suitable microenvironment.^59^ The above results demonstrate that stem cell spheroids wrapped in MPN shells not only manipulate stem cells to ensure survival and stemness maintenance, but also optimize the surrounding inflammatory environment niche. By better understanding how the cellular microenvironment affects cell biology, integration of “seed” and “soil” strategies through stem cell spheroid encapsulation may offer promising therapeutic approaches for tissue repair at sites of complex inflammation.

This study has two major limitations that should be addressed in future research. First, the signaling pathways involved in stemness maintenance upon MPN encapsulation were lack of validation. In this study, we mainly constructed a simple and efficient delivery system for stem cell stemness maintenance in an inflammatory environment. The internal mechanism that how the MPN encapsulation maintains stemness would be explored in our further study. Second, the advantages of the stem cell microsphere capsule proposed in vitro have not been verified in vivo. In the follow-up study, we will continue to establish large animal models to address the in vivo effects of the Fe^III-^TA encapsulation on periodontal tissue regeneration in experimental periodontitis.

## Conclusion

In summary, we proposed a novel strategy for PDLSCs transplantation in a periodontal inflammatory microenvironment, that is, a stem cell microsphere capsule self-assembled with a semipermeable nanoshell based on Fe^III^-TA coordination. In addition to the simplicity and biocompatibility of the strategy, the stem cell microsphere capsule showed several remarkable advantages: (1) cytoprotectability and stemness maintenance: the shell acted as a cytoprotective barrier against multiple stressors from the surrounding environment and maintained cell stemness; (2) remodeling the inflammatory microenvironment: antioxidative, antibacterial and anti-inflammatory properties of the MPN shell could attenuate the damage and remodel the inflammatory injury site. Upon transplantation, the stem cell microsphere capsule could both optimize the cells inside and the microenvironment outside, which proposes a promising method for tissue regeneration in harsh periodontal inflammatory environment. In addition, the encapsulation strategy could be applicable for various cell types and the ability to incorporate multiple polyphenols and metal ions into MPN capsules provides a variety of functional encapsulation materials, which shows great potentials in cell aggregate-based biomedical and biotechnology applications.

## Materials and methods

### Isolation and culture of PDLSCs

This study protocol was approved by the ethical committee of Stomatological Hospital of Shandong University (No. 20210323), and all samples were obtained with informed consents. Periodontal ligament tissues were obtained from healthy donors aged 12–28 and PDLSCs were collected based on our previous study.^60^ Briefly, periodontal ligament tissues were scraped from the middle 1/3 of the root surface of extracted premolars and third molars, cut into tiny pieces, and digested for 40 min in a solution containing collagenase I (3 mg·mL^−1^) and dispase II (4 mg·mL^−1^) at 37 °C. Afterwards, the dissociated cell suspension was passed through a 70-μm filter, and the suspension was plated at a concentration of 60 cells per cm^2^ into petri dishes. The freshly isolated cells were cultured in α-MEM supplemented with 20% fetal bovine serum and 1% penicillin–streptomycin at 37 °C in a humidified atmosphere with 5% CO_2_. The medium was refreshed every 3 days and cells were dissociated with 0.25% trypsin-EDTA solution when reaching 80%–90% confluence. Cells at passage 3–5 were used in the subsequent experiments.

### Fabrication of PDLSC spheroids

For cell spheroid formation, 1 × 10^6^ PDLSCs were dissociated into single cells and seeded into non-adhesive agarose hydrogel molds and incubated in the incubator. The agarose hydrogel molds were made by adding 2% sterile agarose solution to commercial micro-molds (Sigma-Aldrich, USA) and detached from the micro-mold after solidification. After 24 h, cell spheroids were harvested and used in the subsequent experiments.

### Encapsulation of PDLSC spheroids with Fe^III^-TA

Cell spheroids were collected in 1.5 mL centrifuge tubes and washed thrice with phosphate-buffered saline (PBS), and then dispersed in saline solution. The mass ratio of TA to FeCl_3_·6H_2_O in this study was 4:1 as previously reported.^20,23^ Five microliters saline solution of 40 mg·mL^−1^ TA and 5 μL saline solution of 10 mg·mL^−1^ FeCl_3_·6H_2_O were added in sequence to 490 μL cell spheroids suspension with 10 s gently mixing following each addition. Then, 500 μL of 20 mmol·L^−1^ 3-(*N*-morpholino) propanesulfonic acid (MOPS) buffer (pH 7.4) was added to the spheroid@[Fe^III^-TA] suspension to stabilize the pH and form stable Fe^III^-TA shell. Finally, spheroid@[Fe^III^-TA] was washed with saline solution to remove any residual TA and FeCl_3_ and the whole coating process was repeated thrice.

### Spheroid@[Fe^III^-TA] characterization

Scanning electron microscopy (SEM, Hitachi, Japan) was used to examine the surface morphology and element content analysis of cell spheroids after ethanol gradient dehydration and cryogenic freeze-drying. UV–Vis absorption spectra was performed by a ultraviolet-visible spectrophotometer (Shimadzu-UV-2600 I, Japan). FT-IR was obtained by using an infrared spectrometer (Bruker, Germany) in the 400–4 000 cm^−1^ range.

### Histological analysis

Both native spheroid and spheroid@[Fe^III^-TA] were fixed with 4% paraformaldehyde for 15 min and washed thrice with PBS. The cell spheroids were then paraffinized, embedded and sectioned. All paraffin-treated slides were deparaffinized and stained with hematoxylin and eosin and cover-slipped.

### Live and dead cell staining

Both native spheroid and spheroid@[Fe^III^-TA] were incubated in 15 mL centrifuge tubes for 1, 2, and 3 days. Upon staining, cell spheroids were collected and rinsed thrice with PBS. The working solution was prepared according to the manufacturer’s instructions. That is, 5 μL calcein acetoxymethyl (calcein-AM) ester solution and 10 μL propidium iodide (PI) solution (Beyotime Biotechnology, China) were added to 5 mL 1× assay buffer and mixed well. After 30 min incubation with the working solution at 37 °C in the dark, the stained cell spheroids were transferred to 24-well plates and observed under a fluorescence microscope (Leica, Wetzlar, Germany).

### Immunostaining

Cell spheroids were collected and fixed with 4% paraformaldehyde for 15 min. After washing thrice with PBS, cell spheroids were incubated with 2% (wt/v) BSA at 37 °C for 1 h, and then incubated with rabbit anti-CD44 (1: 100, Beyotime, China) overnight at 4 °C. After washing thrice with PBS, samples were incubated with a 1:100 dilution of anti-rabbit IgG for 1 h at room temperature. 4,6-diamino-2-phenylindole dihydrochloride (DAPI) was used to stain the nuclear DNA. Images were viewed and analyzed with CLSM (Andor, England).

### Antibacterial test

#### Antibacterial activity in liquid medium

The antibacterial ability of the Fe^III^-TA shell in liquid medium was tested after culturing *P. gingivalis* in Fe^III^-TA coated 6-well plates. After incubation in the dark at 37 °C for 24 h, the OD value was detected at 600 nm. For the staining of the biofilm, the six-well plate was gently washed thrice with PBS to remove the suspended bacteria after incubation. Then, a N01/PI Double Stain kit (Bestbio, China) was used following the manufacturer’s instruction. Live bacteria were stained with N01 (green) and dead bacteria were stained with PI (red). In this experiment, we only used N01 staining to label the biofilm. After incubation with N01 for 15 min in the dark and washed with PBS, the biofilm was observed under a fluorescence microscope.

#### In situ antibacterial activity

Native spheroid/ spheroid@[Fe^III^-TA] were infected with N01 pre-labeled *P. gingivalis* at a MOI of 1 000. After 4 h of incubation, bacteria inside the cell spheroids were observed under CLSM. After incubation for 24 h, the OD_600_ value of the suspended bacteria was detected and the cell spheroids were collected for RNA isolation.

#### Evaluation of ROS scavenging ability

Here, the radical scavenging ability of the Fe^III^-TA shell was performed by using DPPH and H_2_O_2_ as model radicals. In the DPPH assay, native spheroid and spheroid@[Fe^III^-TA] were added in 12-well plates and then 0.1 mmol·L^−1^ DPPH/ethanol solution was added into each well. After incubation at 37 °C for 1 h, the supernatant was collected, and the absorbance was measured at 570 nm with an UV spectrophotometer. For the H_2_O_2_ assay, native spheroid and spheroid@[Fe^III^-TA] were collected in 1.5 mL centrifuge tubes, then incubated with 0.5 mL of H_2_O_2_ in aqueous solution (50 mmol·L^−1^) at 37 °C for 2 h. Afterwards, a hydrogen peroxide assay kit was used to detect the residual concentration of H_2_O_2_ (Nanjing Jiancheng Bioengineering Institute, China). The eliminated H_2_O_2_ was also calculated. Native spheroid and spheroid@[Fe^III^-TA] were incubated with H_2_O_2_ (300 μmol·L^−1^) for 2 h for intracellular ROS detection and 24 h for mRNA isolation.

#### Intracellular ROS measurement

ROS assay kit (Bestbio, China) was used to detect the intracellular ROS level. Native spheroid and spheroid@[Fe^III^-TA] were collected following H_2_O_2_ stimulation and incubated with DCFH-DA at 37 °C for 20 min with gentle shaking every 5 min. After washing with PBS, cell spheroids were immediately examined for fluorescence under a fluorescence microscope or flow cytometry. For flow cytometric analysis, after incubation with DCFH-DA and washing with PBS, native spheroid and spheroid@[Fe^III^-TA] were collected and trypsinized into single cells, then flow cytometry was used to detect the fluorescence intensity of the cell spheroid-derived cells.

#### Cell apoptosis analysis

One Step TUNEL Apoptosis Assay Kit (Elabscience Biotechnology, China) was used to detect the cell apoptosis according to the manufacturer’s protocol. Native spheroid and spheroid@[Fe^III^-TA] were collected following H_2_O_2_ stimulation and fixed with 4% paraformaldehyde for 20 min. After washed twice with PBS, cell spheroids were permeabilized with 0.2% Trition X-100 at 37 °C for 10 min and rinsed with PBS. Then cell spheroids were incubated with TdT Equilibration Buffer at 37 °C for 20 min and incubated with labeling working buffer at 37 °C for 1 h in the dark. After washed twice with PBS, DAPI was used to stain the nuclear DNA. After incubation for 5 min and washing with PBS, the cell spheroids were immediately examined for fluorescence under a fluorescence microscope.

#### Quantitative real-time polymerase chain reaction (qRT-PCR) analysis

A RaPure Total RNA Micro Kit (Magen, China) was used to isolate total RNA following the manufacturer’s instructions. cDNA was obtained by reverse transcription using Evo M-MLV RT Kit (Accurate Biology, China). qRT-PCR were performed with SYBR Green Premix *Pro Taq* HS qPCR Kit (Accurate Biology, China) in a LightCycler 96 Real-Time PCR System (Roche, Basel, Switzerland). The primer sequences were as follows: *GAPDH*, 5′-GCACCGTCAAGGCTGAGAAC-3′ and 5′-TGGTGAAGACGCCAGTGGA; *Sox2*, 5′-CATGAAGGAGCACCCGGATT-3′ and 5′-GTTCATGTGCGCGTAACTGT-3′; *Nanog*, 5′-AATGGTGTGACGCAGGGATG-3′ and 5′-TGCACCAGGTCTGAGTGTTC-3′; *IL-6*, 5′-ACTCACCTCTTCAGAACGAATTG-3′ and 5′-CCATCTTTGGAAGGTTCAGGTTG-3′; *IL-8*, 5′-ACTGAGAGTGATTGAGAGTGGAC-3′ and 5′-AACCCTCTGCACCCAGTTTTC-3′.

#### Enzyme-linked immunosorbent assay (ELISA)

Conditioned medium was collected from native spheroid and spheroid@[Fe^III^-TA] cultures after 6 h stimulation of LPS (100 ng·mL^−^^1^, Beyotime, China). Protein levels of *IL-6* and *IL-8* were determined by using ELISA kits (Biolegend, USA) following the manufacturer’s instructions.

#### RNA-sequencing analysis

Total RNAs from native spheroid and spheroid@[Fe^III^-TA] were isolated using Trizol reagent. Quantity and purity of the total RNA were analyzed with Bioanalyzer 2100 and RNA 6000 Nano LabChip Kit (Agilent, USA). Following the vendor’s recommended protocol, RNA-sequencing analysis was performed on an Illumina NovaseqTM 6000 (LC-Bio Technology CO., Ltd, China).

#### Statistical analysis

All the quantitative data were presented as mean ± standard deviation (SD). The difference between experimental groups was analyzed by GraphPad Prism 5 (GraphPad Software) using one-way analysis of variance (ANOVA) followed by either Tukey’s honest significant difference test (for more than two variables) or Student’s *t*-test (for two variables). A difference was considered statistically significant if the *P* value was less than 0.05.

## Supplementary information

Supplemental material

## Data Availability

All data are included in the manuscript.
