# Supplementary material for: Stem cell microencapsulation maintains stemness in inflammatory microenvironment
Source: Int J Oral Sci. 2022 Oct 10;14:48. doi: 10.1038/s41368-022-00198-w (PMC9551082; doi:10.1038/s41368-022-00198-w)
Supplement: Supplementary file 1 — Supplemental material [file 41368_2022_198_MOESM1_ESM.docx]

**
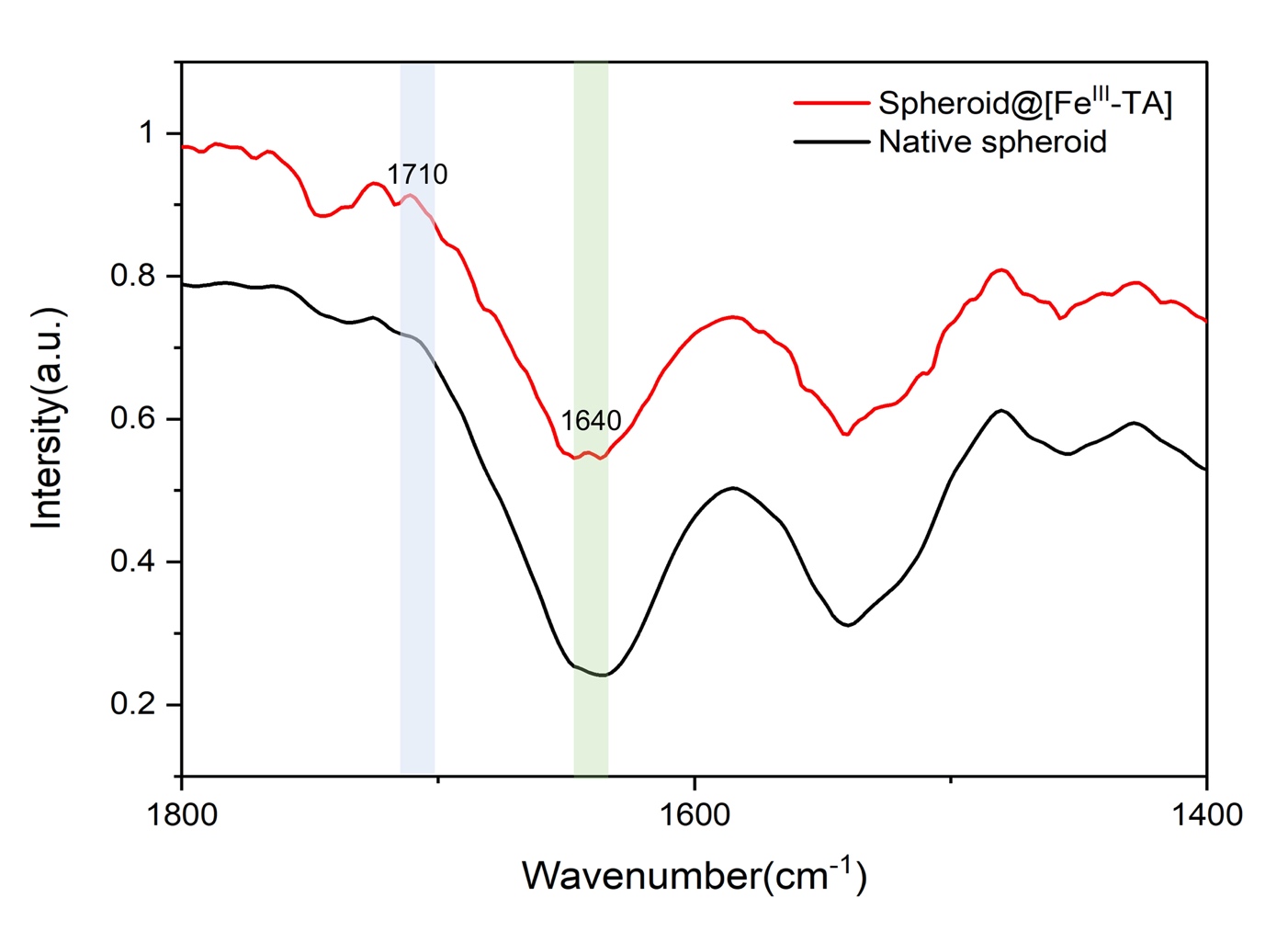
Figure S1**

**Figure S1.** FI-TR spectra of native spheroid and spheroid@[Fe^III^-TA].

**
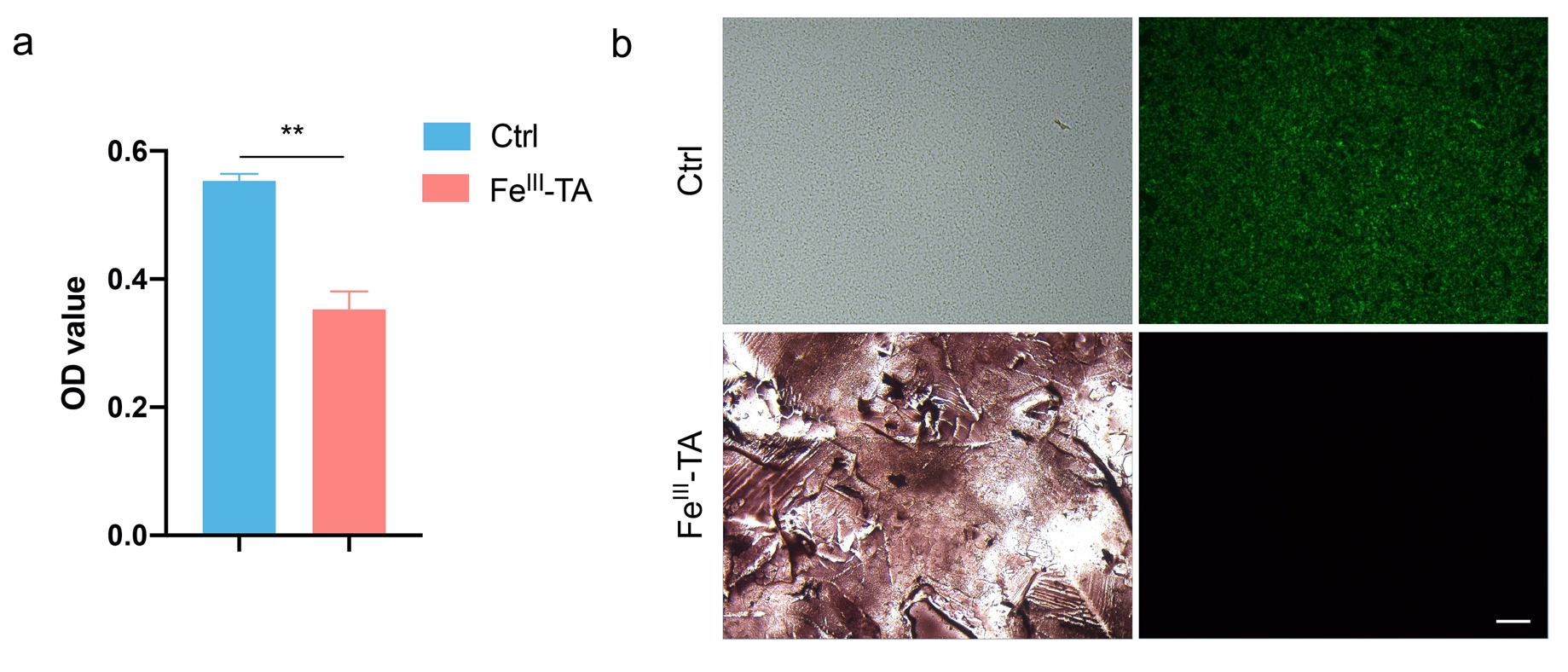
Figure S2**

**Figure S2**. Inhibitory effect of the Fe^III^-TA shell on bacteria. (a) Optical density values of *P. gingivalis* after incubation with Fe^III^-TA for 24 h. (b) Fluorescence images of biofilm stained with N01. Scale bar = 50 μm. ^**^*P* < 0.01.

**
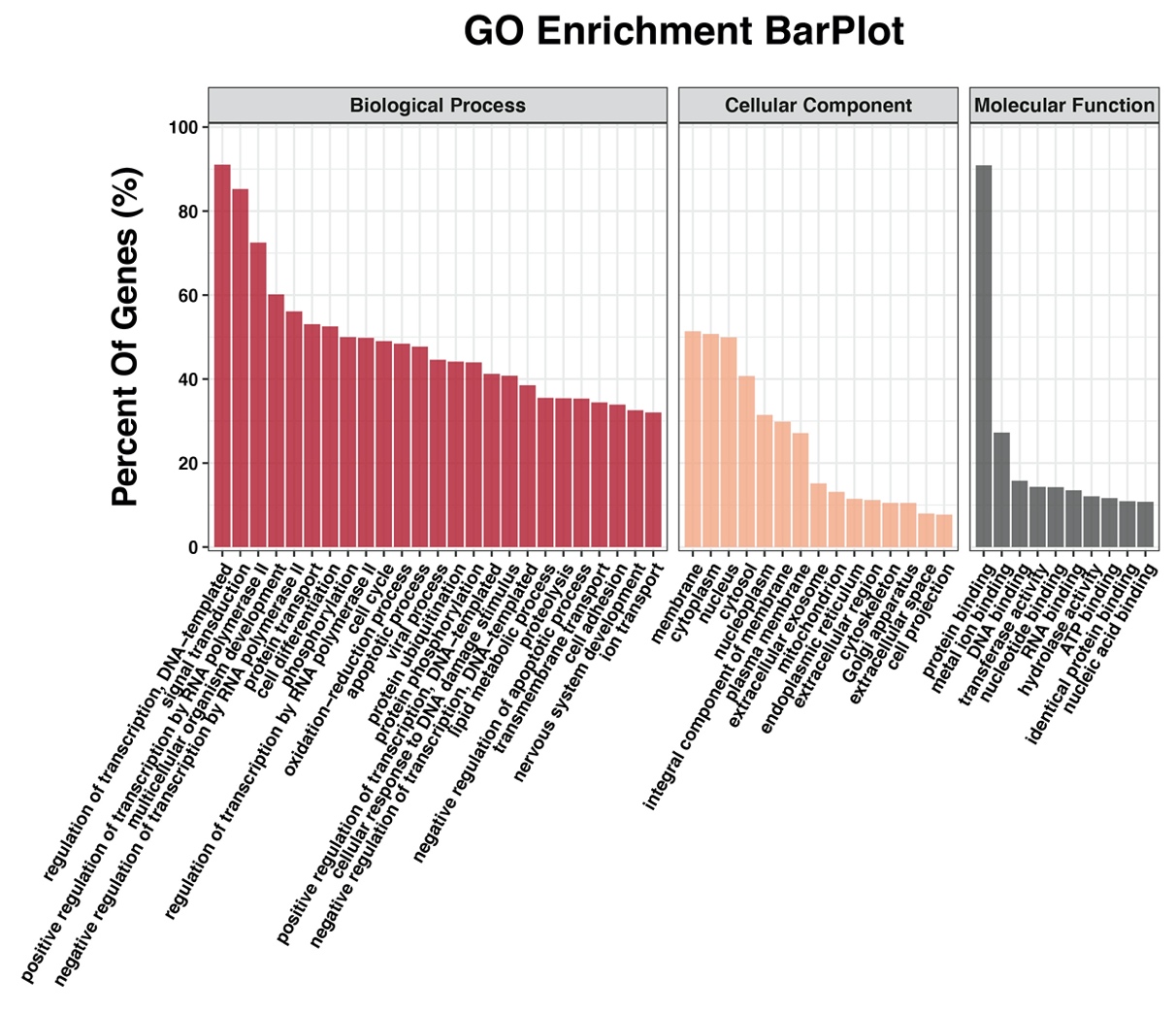
Figure S3**

**Figure S3**. Histogram depiction of GO functional enrichment results of DEGs upon encapsulation with the Fe^III^-TA shell.

| **Table S1.** KEGG pathways enriched results of DEGs upon encapsulation with the Fe^III^-TA shell. | | | | | | | |
| --- | --- | --- | --- | --- | --- | --- | --- |
| **ID** | **Description** | **GeneRatio** | **BgRatio** | **pvalue** | **p.adjust** | **qvalue** | **Count** |
| **hsa05014** | Amyotrophic lateral sclerosis | 315/5385 | 364/8114 | 2.80818365837602E-19 | 9.32316974580837E-17 | 4.84781178919649E-17 | 315 |
| **hsa05016** | Huntington disease | 269/5385 | 306/8114 | 1.09346883081737E-18 | 1.81515825915683E-16 | 9.43836253968674E-17 | 269 |
| **hsa04144** | Endocytosis | 223/5385 | 251/8114 | 6.32255276988974E-17 | 6.99695839867798E-15 | 3.63824089214708E-15 | 223 |
| **hsa04141** | Protein processing in endoplasmic reticulum | 158/5385 | 171/8114 | 3.81711440797241E-16 | 3.1682049586171E-14 | 1.64738621817757E-14 | 158 |
| **hsa04120** | Ubiquitin mediated proteolysis | 134/5385 | 142/8114 | 5.53075751470114E-16 | 3.67242298976156E-14 | 1.90956680507576E-14 | 134 |
| **hsa04714** | Thermogenesis | 206/5385 | 232/8114 | 1.17975768338952E-15 | 6.52799251475532E-14 | 3.39439052764703E-14 | 206 |
| **hsa05012** | Parkinson disease | 232/5385 | 266/8114 | 2.43438389612252E-15 | 1.15459350501811E-13 | 6.0035933678811E-14 | 232 |
| **hsa03040** | Spliceosome | 137/5385 | 147/8114 | 5.36531491557476E-15 | 2.22660568996353E-13 | 1.15777848178192E-13 | 137 |
| **hsa05132** | Salmonella infection | 218/5385 | 249/8114 | 6.80281888724553E-15 | 2.50948430062835E-13 | 1.3048681842202E-13 | 218 |
| **hsa05208** | Chemical carcinogenesis - reactive oxygen species | 197/5385 | 223/8114 | 1.75794853226641E-14 | 5.83638912712448E-13 | 3.03477430833359E-13 | 197 |
| **hsa05010** | Alzheimer disease | 320/5385 | 384/8114 | 1.96600680248025E-14 | 5.93376598566767E-13 | 3.08540780484939E-13 | 320 |
| **hsa04140** | Autophagy - animal | 131/5385 | 141/8114 | 4.29615980346544E-14 | 1.1886042122921E-12 | 6.18044041902045E-13 | 131 |
| **hsa04932** | Non-alcoholic fatty liver disease | 142/5385 | 155/8114 | 8.28851062873683E-14 | 2.11675809903125E-12 | 1.10066052073914E-12 | 142 |
| **hsa05131** | Shigellosis | 214/5385 | 247/8114 | 1.49132239063125E-13 | 3.5365645263541E-12 | 1.83892385010169E-12 | 214 |
| **hsa05022** | Pathways of neurodegeneration - multiple diseases | 384/5385 | 476/8114 | 8.44572892167096E-13 | 1.86932133466317E-11 | 9.71999679406342E-12 | 384 |
| **hsa05020** | Prion disease | 230/5385 | 273/8114 | 1.13527294500476E-11 | 2.35569136088488E-10 | 1.22489975645251E-10 | 230 |
| **hsa03013** | Nucleocytoplasmic transport | 101/5385 | 108/8114 | 1.28173123634913E-11 | 2.50314570863477E-10 | 1.30157227715949E-10 | 101 |
| **hsa05220** | Chronic myeloid leukemia | 74/5385 | 76/8114 | 1.94667155201515E-11 | 3.59052752927238E-10 | 1.86698324286833E-10 | 74 |
| **hsa04218** | Cellular senescence | 139/5385 | 156/8114 | 3.22544921225009E-11 | 5.6360480971949E-10 | 2.93060205434357E-10 | 139 |
| **hsa04110** | Cell cycle | 115/5385 | 126/8114 | 3.93786916133808E-11 | 6.53686280782121E-10 | 3.39900285504971E-10 | 115 |
| **hsa05210** | Colorectal cancer | 82/5385 | 86/8114 | 6.33695799694136E-11 | 1.00184288332597E-09 | 5.20932887969114E-10 | 82 |
| **hsa00190** | Oxidative phosphorylation | 121/5385 | 134/8114 | 7.52249125165486E-11 | 1.13521231615883E-09 | 5.90281610177702E-10 | 121 |
| **hsa05212** | Pancreatic cancer | 73/5385 | 76/8114 | 2.53512473043205E-10 | 3.65939743697149E-09 | 1.90279384801307E-09 | 73 |
| **hsa04150** | mTOR signaling pathway | 136/5385 | 156/8114 | 1.8180031587375E-09 | 2.51490436958687E-08 | 1.30768648260066E-08 | 136 |
| **hsa05415** | Diabetic cardiomyopathy | 172/5385 | 203/8114 | 1.93923327218471E-09 | 2.57530178546129E-08 | 1.33909160689807E-08 | 172 |
| **hsa04142** | Lysosome | 117/5385 | 132/8114 | 2.63048610824941E-09 | 3.35892841514924E-08 | 1.74655757794697E-08 | 117 |
| **hsa05211** | Renal cell carcinoma | 66/5385 | 69/8114 | 3.4553819531622E-09 | 4.24233625291051E-08 | 2.20590724628194E-08 | 66 |
| **hsa03010** | Ribosome | 137/5385 | 158/8114 | 3.57787394823778E-09 | 4.24233625291051E-08 | 2.20590724628194E-08 | 137 |
| **hsa05203** | Viral carcinogenesis | 172/5385 | 204/8114 | 4.28720019738421E-09 | 4.9081050535571E-08 | 2.55209013564795E-08 | 172 |
| **hsa04520** | Adherens junction | 67/5385 | 71/8114 | 1.47852312387258E-08 | 1.63623225708566E-07 | 8.50799271281065E-08 | 67 |
| **hsa05168** | Herpes simplex virus 1 infection | 382/5385 | 495/8114 | 4.20518775059051E-08 | 4.41939329083215E-07 | 2.29797241501735E-07 | 382 |
| **hsa01521** | EGFR tyrosine kinase inhibitor resistance | 73/5385 | 79/8114 | 4.2596561839346E-08 | 4.41939329083215E-07 | 2.29797241501735E-07 | 73 |
| **hsa04211** | Longevity regulating pathway | 81/5385 | 89/8114 | 4.70948719762604E-08 | 4.73802954427832E-07 | 2.46365518472303E-07 | 81 |
| **hsa05166** | Human T-cell leukemia virus 1 infection | 183/5385 | 222/8114 | 4.88248616755772E-08 | 4.76760414008577E-07 | 2.47903322439463E-07 | 183 |
| **hsa05205** | Proteoglycans in cancer | 170/5385 | 205/8114 | 6.36206180042452E-08 | 6.0348700506884E-07 | 3.13797935419435E-07 | 170 |
| **hsa05225** | Hepatocellular carcinoma | 142/5385 | 168/8114 | 7.11534448984605E-08 | 6.56192880730247E-07 | 3.4120365389905E-07 | 142 |
| **hsa04137** | Mitophagy - animal | 67/5385 | 72/8114 | 7.4449368205959E-08 | 6.68032168766984E-07 | 3.47359783379155E-07 | 67 |
| **hsa05135** | Yersinia infection | 118/5385 | 137/8114 | 1.02996444359416E-07 | 8.99863671771742E-07 | 4.67906284624495E-07 | 118 |
| **hsa04068** | FoxO signaling pathway | 113/5385 | 131/8114 | 1.61971338959069E-07 | 1.37883293677977E-06 | 7.16958153557014E-07 | 113 |
| **hsa03018** | RNA degradation | 72/5385 | 79/8114 | 2.35385519355064E-07 | 1.95369981064703E-06 | 1.01587434669028E-06 | 72 |
| **hsa05161** | Hepatitis B | 136/5385 | 162/8114 | 3.15937417749971E-07 | 2.50552361588171E-06 | 1.30280872861319E-06 | 136 |
| **hsa04115** | p53 signaling pathway | 67/5385 | 73/8114 | 3.16963830924794E-07 | 2.50552361588171E-06 | 1.30280872861319E-06 | 67 |
| **hsa04722** | Neurotrophin signaling pathway | 103/5385 | 119/8114 | 3.98585505869617E-07 | 3.0774508825282E-06 | 1.60019640055366E-06 | 103 |
| **hsa04668** | TNF signaling pathway | 97/5385 | 112/8114 | 8.2145222839468E-07 | 6.1982304506144E-06 | 3.222922618582E-06 | 97 |
| **hsa04210** | Apoptosis | 115/5385 | 136/8114 | 1.21764709404609E-06 | 8.98352967162895E-06 | 4.67120756546337E-06 | 115 |
| **hsa05213** | Endometrial cancer | 54/5385 | 58/8114 | 1.43141384217157E-06 | 1.03310738174122E-05 | 5.3718963413304E-06 | 54 |
| **hsa00562** | Inositol phosphate metabolism | 66/5385 | 73/8114 | 1.61169652463166E-06 | 1.13847499186747E-05 | 5.91978118789682E-06 | 66 |
| **hsa05017** | Spinocerebellar ataxia | 120/5385 | 143/8114 | 1.64970624739161E-06 | 1.14104682111253E-05 | 5.93315404763649E-06 | 120 |
| **hsa05100** | Bacterial invasion of epithelial cells | 69/5385 | 77/8114 | 2.08784946741833E-06 | 1.41462453710793E-05 | 7.35568877887446E-06 | 69 |
| **hsa04012** | ErbB signaling pathway | 75/5385 | 85/8114 | 3.11064395618209E-06 | 2.06546758690491E-05 | 1.07399075539761E-05 | 75 |
| **hsa04360** | Axon guidance | 148/5385 | 182/8114 | 4.57422793958259E-06 | 2.93983960078441E-05 | 1.52864202450426E-05 | 148 |
| **hsa03015** | mRNA surveillance pathway | 84/5385 | 97/8114 | 4.60456804942137E-06 | 2.93983960078441E-05 | 1.52864202450426E-05 | 84 |
| **hsa05222** | Small cell lung cancer | 80/5385 | 92/8114 | 5.4620672115663E-06 | 3.42152134762267E-05 | 1.77910431518744E-05 | 80 |
| **hsa04933** | AGE-RAGE signaling pathway in diabetic complications | 86/5385 | 100/8114 | 6.60605308540357E-06 | 4.06149930435923E-05 | 2.1118766198951E-05 | 86 |
| **hsa00280** | Valine, leucine and isoleucine degradation | 45/5385 | 48/8114 | 6.95703761520216E-06 | 4.12201476127599E-05 | 2.14334312254047E-05 | 45 |
| **hsa04910** | Insulin signaling pathway | 114/5385 | 137/8114 | 6.98496769717926E-06 | 4.12201476127599E-05 | 2.14334312254047E-05 | 114 |
| **hsa01524** | Platinum drug resistance | 65/5385 | 73/8114 | 7.07695305399794E-06 | 4.12201476127599E-05 | 2.14334312254047E-05 | 65 |
| **hsa03030** | DNA replication | 35/5385 | 36/8114 | 7.24527362567512E-06 | 4.14729455814507E-05 | 2.15648797570004E-05 | 35 |
| **hsa04071** | Sphingolipid signaling pathway | 100/5385 | 119/8114 | 1.08088683775847E-05 | 6.08227847687819E-05 | 3.1626305333165E-05 | 100 |
| **hsa05231** | Choline metabolism in cancer | 84/5385 | 98/8114 | 1.12658944489702E-05 | 6.23379492843016E-05 | 3.24141524496686E-05 | 84 |
| **hsa04010** | MAPK signaling pathway | 228/5385 | 294/8114 | 1.33977475584552E-05 | 7.29188883509365E-05 | 3.79159723828585E-05 | 228 |
| **hsa03050** | Proteasome | 43/5385 | 46/8114 | 1.40128438895609E-05 | 7.50365188924875E-05 | 3.90170865515788E-05 | 43 |
| **hsa05163** | Human cytomegalovirus infection | 178/5385 | 225/8114 | 1.44493476877745E-05 | 7.61367609402914E-05 | 3.95891845092194E-05 | 178 |
| **hsa05215** | Prostate cancer | 83/5385 | 97/8114 | 1.46769659643935E-05 | 7.61367609402914E-05 | 3.95891845092194E-05 | 83 |
| **hsa05214** | Glioma | 66/5385 | 75/8114 | 1.52893178292722E-05 | 7.80931310664363E-05 | 4.0606447352237E-05 | 66 |
| **hsa03020** | RNA polymerase | 33/5385 | 34/8114 | 1.5644990560538E-05 | 7.86990434257367E-05 | 4.09215064103387E-05 | 33 |
| **hsa05120** | Epithelial cell signaling in Helicobacter pylori infection | 62/5385 | 70/8114 | 1.73893897681954E-05 | 8.61683194483713E-05 | 4.48053404867879E-05 | 62 |
| **hsa05165** | Human papillomavirus infection | 254/5385 | 331/8114 | 1.80008436757024E-05 | 8.78864720637234E-05 | 4.5698736266489E-05 | 254 |
| **hsa04152** | AMPK signaling pathway | 100/5385 | 120/8114 | 2.26277000009169E-05 | 1.08875310149339E-04 | 5.66123996971833E-05 | 100 |
| **hsa04136** | Autophagy - other | 31/5385 | 32/8114 | 3.36699011971798E-05 | 1.59691531392339E-04 | 8.30355458095863E-05 | 31 |
| **hsa04914** | Progesterone-mediated oocyte maturation | 86/5385 | 102/8114 | 3.46593626621972E-05 | 1.62069132448584E-04 | 8.42718380518954E-05 | 86 |
| **hsa05223** | Non-small cell lung cancer | 63/5385 | 72/8114 | 3.63055435046134E-05 | 1.67408895049051E-04 | 8.70483791631082E-05 | 63 |
| **hsa04919** | Thyroid hormone signaling pathway | 100/5385 | 121/8114 | 4.55143041451729E-05 | 2.06996561317773E-04 | 1.07632961496876E-04 | 100 |
| **hsa04213** | Longevity regulating pathway - multiple species | 55/5385 | 62/8114 | 4.78577604947416E-05 | 2.14713195733165E-04 | 1.11645415663408E-04 | 55 |
| **hsa03420** | Nucleotide excision repair | 43/5385 | 47/8114 | 5.84382550112934E-05 | 2.58686675516659E-04 | 1.34510509780381E-04 | 43 |
| **hsa05170** | Human immunodeficiency virus 1 infection | 166/5385 | 212/8114 | 7.95203381558929E-05 | 3.47378319312585E-04 | 1.80627914924743E-04 | 166 |
| **hsa04510** | Focal adhesion | 158/5385 | 201/8114 | 8.1445253051142E-05 | 3.51166545623106E-04 | 1.82597696519307E-04 | 158 |
| **hsa01522** | Endocrine resistance | 82/5385 | 98/8114 | 9.27175287357723E-05 | 3.94643840259954E-04 | 2.05204786945569E-04 | 82 |
| **hsa03022** | Basal transcription factors | 41/5385 | 45/8114 | 1.12328076515915E-04 | 4.72062296244098E-04 | 2.45460420367889E-04 | 41 |
| **hsa04070** | Phosphatidylinositol signaling system | 81/5385 | 97/8114 | 1.18039996234976E-04 | 4.89865984375152E-04 | 2.54717886612317E-04 | 81 |
| **hsa05169** | Epstein-Barr virus infection | 158/5385 | 202/8114 | 1.30017861146014E-04 | 5.32912714820701E-04 | 2.771010945802E-04 | 158 |
| **hsa05167** | Kaposi sarcoma-associated herpesvirus infection | 152/5385 | 194/8114 | 1.49265054639543E-04 | 6.04341440735711E-04 | 3.14242220293775E-04 | 152 |
| **hsa03410** | Base excision repair | 31/5385 | 33/8114 | 1.99650442685832E-04 | 7.89094606805909E-04 | 4.10309180457099E-04 | 31 |
| **hsa04130** | SNARE interactions in vesicular transport | 31/5385 | 33/8114 | 1.99650442685832E-04 | 7.89094606805909E-04 | 4.10309180457099E-04 | 31 |
| **hsa05418** | Fluid shear stress and atherosclerosis | 111/5385 | 139/8114 | 2.98238798852219E-04 | 0.001164886 | 6.05710997049707E-04 | 111 |
| **hsa01250** | Biosynthesis of nucleotide sugars | 34/5385 | 37/8114 | 3.03084827503179E-04 | 0.001170048 | 6.08395492172844E-04 | 34 |
| **hsa04390** | Hippo signaling pathway | 124/5385 | 157/8114 | 3.24966344671749E-04 | 0.001235874 | 6.42623374060796E-04 | 124 |
| **hsa00563** | Glycosylphosphatidylinositol (GPI)-anchor biosynthesis | 25/5385 | 26/8114 | 3.27581183362699E-04 | 0.001235874 | 6.42623374060796E-04 | 25 |
| **hsa04931** | Insulin resistance | 88/5385 | 108/8114 | 3.4840538047218E-04 | 0.00129967 | 6.75795179153609E-04 | 88 |
| **hsa00310** | Lysine degradation | 54/5385 | 63/8114 | 4.37670859017988E-04 | 0.001614519 | 8.39509016128071E-04 | 54 |
| **hsa00520** | Amino sugar and nucleotide sugar metabolism | 43/5385 | 49/8114 | 5.80613470753574E-04 | 0.002118282 | 0.001101453 | 43 |
| **hsa04350** | TGF-beta signaling pathway | 77/5385 | 94/8114 | 5.91331155117128E-04 | 0.002133934 | 0.001109592 | 77 |
| **hsa04114** | Oocyte meiosis | 104/5385 | 131/8114 | 6.87359289228709E-04 | 0.002453799 | 0.001275913 | 104 |
| **hsa01212** | Fatty acid metabolism | 49/5385 | 57/8114 | 7.02299486741452E-04 | 0.002480462 | 0.001289777 | 49 |
| **hsa05235** | PD-L1 expression and PD-1 checkpoint pathway in cancer | 73/5385 | 89/8114 | 7.5870900218937E-04 | 0.002651488 | 0.001378707 | 73 |
| **hsa04935** | Growth hormone synthesis, secretion and action | 95/5385 | 119/8114 | 8.18027760757968E-04 | 0.002829013 | 0.001471015 | 95 |
| **hsa03060** | Protein export | 22/5385 | 23/8114 | 0.001004427 | 0.003369958 | 0.001752293 | 22 |
| **hsa03430** | Mismatch repair | 22/5385 | 23/8114 | 0.001004427 | 0.003369958 | 0.001752293 | 22 |
| **hsa04926** | Relaxin signaling pathway | 102/5385 | 129/8114 | 0.001004897 | 0.003369958 | 0.001752293 | 102 |
| **hsa04370** | VEGF signaling pathway | 50/5385 | 59/8114 | 0.001247803 | 0.004142706 | 0.002154102 | 50 |
| **hsa00510** | N-Glycan biosynthesis | 43/5385 | 50/8114 | 0.001481342 | 0.004821623 | 0.002507122 | 43 |
| **hsa05110** | Vibrio cholerae infection | 43/5385 | 50/8114 | 0.001481342 | 0.004821623 | 0.002507122 | 43 |
| **hsa00640** | Propanoate metabolism | 29/5385 | 32/8114 | 0.001559618 | 0.004981996 | 0.002590511 | 29 |
| **hsa03460** | Fanconi anemia pathway | 46/5385 | 54/8114 | 0.001560625 | 0.004981996 | 0.002590511 | 46 |
| **hsa00240** | Pyrimidine metabolism | 49/5385 | 58/8114 | 0.001610944 | 0.005093651 | 0.002648569 | 49 |
| **hsa01200** | Carbon metabolism | 91/5385 | 115/8114 | 0.001773492 | 0.005554712 | 0.002888309 | 91 |
| **hsa04710** | Circadian rhythm | 28/5385 | 31/8114 | 0.002147802 | 0.006664209 | 0.00346522 | 28 |
| **hsa05171** | Coronavirus disease - COVID-19 | 174/5385 | 232/8114 | 0.002467548 | 0.007585426 | 0.003944229 | 174 |
| **hsa05130** | Pathogenic Escherichia coli infection | 149/5385 | 197/8114 | 0.002779087 | 0.008464741 | 0.004401451 | 149 |
| **hsa00020** | Citrate cycle (TCA cycle) | 27/5385 | 30/8114 | 0.002949258 | 0.008901397 | 0.004628501 | 27 |
| **hsa00532** | Glycosaminoglycan biosynthesis - chondroitin sulfate / dermatan sulfate | 19/5385 | 20/8114 | 0.003033609 | 0.009073496 | 0.004717988 | 19 |
| **hsa04015** | Rap1 signaling pathway | 158/5385 | 210/8114 | 0.00306506 | 0.009085713 | 0.00472434 | 158 |
| **hsa05221** | Acute myeloid leukemia | 55/5385 | 67/8114 | 0.003258412 | 0.009573388 | 0.004977919 | 55 |
| **hsa04666** | Fc gamma R-mediated phagocytosis | 77/5385 | 97/8114 | 0.003346052 | 0.009744644 | 0.005066968 | 77 |
| **hsa05230** | Central carbon metabolism in cancer | 57/5385 | 70/8114 | 0.003939258 | 0.011372467 | 0.005913395 | 57 |
| **hsa01240** | Biosynthesis of cofactors | 117/5385 | 153/8114 | 0.004040685 | 0.01156472 | 0.006013361 | 117 |
| **hsa04810** | Regulation of actin cytoskeleton | 163/5385 | 218/8114 | 0.004130227 | 0.011719959 | 0.006094082 | 163 |
| **hsa05216** | Thyroid cancer | 32/5385 | 37/8114 | 0.005120682 | 0.014407343 | 0.007491453 | 32 |
| **hsa04066** | HIF-1 signaling pathway | 85/5385 | 109/8114 | 0.005317782 | 0.014806895 | 0.00769921 | 85 |
| **hsa03440** | Homologous recombination | 35/5385 | 41/8114 | 0.005396489 | 0.014806895 | 0.00769921 | 35 |
| **hsa05219** | Bladder cancer | 35/5385 | 41/8114 | 0.005396489 | 0.014806895 | 0.00769921 | 35 |
| **hsa05218** | Melanoma | 58/5385 | 72/8114 | 0.005779495 | 0.015727805 | 0.00817806 | 58 |
| **hsa04215** | Apoptosis - multiple species | 28/5385 | 32/8114 | 0.006265092 | 0.016910654 | 0.008793111 | 28 |
| **hsa01230** | Biosynthesis of amino acids | 60/5385 | 75/8114 | 0.006722811 | 0.017999784 | 0.009359431 | 60 |
| **hsa04912** | GnRH signaling pathway | 73/5385 | 93/8114 | 0.00711655 | 0.018901558 | 0.009828331 | 73 |
| **hsa01040** | Biosynthesis of unsaturated fatty acids | 24/5385 | 27/8114 | 0.007490472 | 0.019736799 | 0.010262635 | 24 |
| **hsa04928** | Parathyroid hormone synthesis, secretion and action | 82/5385 | 106/8114 | 0.008934143 | 0.023286911 | 0.012108603 | 82 |
| **hsa00230** | Purine metabolism | 97/5385 | 127/8114 | 0.008978086 | 0.023286911 | 0.012108603 | 97 |
| **hsa04540** | Gap junction | 69/5385 | 88/8114 | 0.009188206 | 0.023647165 | 0.012295926 | 69 |
| **hsa04310** | Wnt signaling pathway | 125/5385 | 167/8114 | 0.010583836 | 0.027029488 | 0.014054648 | 125 |
| **hsa00770** | Pantothenate and CoA biosynthesis | 19/5385 | 21/8114 | 0.011877382 | 0.030101458 | 0.015651994 | 19 |
| **hsa04625** | C-type lectin receptor signaling pathway | 80/5385 | 104/8114 | 0.012476823 | 0.0313811 | 0.016317376 | 80 |
| **hsa04072** | Phospholipase D signaling pathway | 111/5385 | 148/8114 | 0.014026081 | 0.035012474 | 0.018205598 | 111 |
| **hsa04146** | Peroxisome | 64/5385 | 82/8114 | 0.014234062 | 0.035266481 | 0.018337675 | 64 |
| **hsa04392** | Hippo signaling pathway - multiple species | 25/5385 | 29/8114 | 0.014705598 | 0.036164878 | 0.018804819 | 25 |
| **hsa00051** | Fructose and mannose metabolism | 28/5385 | 33/8114 | 0.015149715 | 0.036983128 | 0.019230288 | 28 |
| **hsa04340** | Hedgehog signaling pathway | 45/5385 | 56/8114 | 0.015728463 | 0.038115692 | 0.019819193 | 45 |
| **hsa00920** | Sulfur metabolism | 10/5385 | 10/8114 | 0.016530471 | 0.039768959 | 0.02067885 | 10 |
| **hsa05142** | Chagas disease | 78/5385 | 102/8114 | 0.017252846 | 0.041208236 | 0.021427238 | 78 |
| **hsa04917** | Prolactin signaling pathway | 55/5385 | 70/8114 | 0.017847588 | 0.042324281 | 0.022007553 | 55 |
| **hsa00604** | Glycosphingolipid biosynthesis - ganglio series | 14/5385 | 15/8114 | 0.018280594 | 0.042822241 | 0.022266479 | 14 |
| **hsa04962** | Vasopressin-regulated water reabsorption | 36/5385 | 44/8114 | 0.018315537 | 0.042822241 | 0.022266479 | 36 |
| **hsa04936** | Alcoholic liver disease | 106/5385 | 142/8114 | 0.020063974 | 0.046582094 | 0.024221507 | 106 |

**Table S2.** KEGG pathways enriched results of upregulated DEGs upon encapsulation with the Fe^III^-TA shell.

| **ID** | **Description** | **GeneRatio** | **BgRatio** | **pvalue** | **p.adjust** | **qvalue** | **Count** |
| --- | --- | --- | --- | --- | --- | --- | --- |
| **hsa04120** | Ubiquitin mediated proteolysis | 134/4907 | 142/8114 | 6.47589220717955E-21 | 1.27269575669122E-18 | 6.73875051894207E-19 | 134 |
| **hsa04141** | Protein processing in endoplasmic reticulum | 157/4907 | 171/8114 | 7.6668419077784E-21 | 1.27269575669122E-18 | 6.73875051894207E-19 | 157 |
| **hsa05016** | Huntington disease | 257/4907 | 306/8114 | 8.14346558939944E-20 | 9.01210191893538E-18 | 4.77178509975336E-18 | 257 |
| **hsa05208** | Chemical carcinogenesis - reactive oxygen species | 195/4907 | 223/8114 | 2.04969936196124E-19 | 1.45349660675265E-17 | 7.69606637056732E-18 | 195 |
| **hsa05014** | Amyotrophic lateral sclerosis | 298/4907 | 364/8114 | 2.18900091378412E-19 | 1.45349660675265E-17 | 7.69606637056732E-18 | 298 |
| **hsa04714** | Thermogenesis | 201/4907 | 232/8114 | 6.25454826704348E-19 | 3.46085004109739E-17 | 1.83247291332677E-17 | 201 |
| **hsa04140** | Autophagy - animal | 130/4907 | 141/8114 | 7.41425495707164E-18 | 3.51647520821112E-16 | 1.86192568094882E-16 | 130 |
| **hsa05012** | Parkinson disease | 223/4907 | 266/8114 | 3.8895066287664E-17 | 1.61414525093805E-15 | 8.54667903952616E-16 | 223 |
| **hsa04144** | Endocytosis | 211/4907 | 251/8114 | 1.50941869583825E-16 | 5.56807785575889E-15 | 2.94822131233904E-15 | 211 |
| **hsa04932** | Non-alcoholic fatty liver disease | 139/4907 | 155/8114 | 2.60603351567764E-16 | 8.65203127204977E-15 | 4.58113260124386E-15 | 139 |
| **hsa05010** | Alzheimer disease | 303/4907 | 384/8114 | 2.6104404458046E-15 | 7.8787838909739E-14 | 4.17170865501788E-14 | 303 |
| **hsa05132** | Salmonella infection | 207/4907 | 249/8114 | 3.51963186901899E-15 | 9.73764817095254E-14 | 5.15595194847519E-14 | 207 |
| **hsa00190** | Oxidative phosphorylation | 121/4907 | 134/8114 | 6.65216910506529E-15 | 1.69886164837052E-13 | 8.99524081413688E-14 | 121 |
| **hsa03040** | Spliceosome | 130/4907 | 147/8114 | 3.39387600122171E-14 | 8.04833451718291E-13 | 4.26148340003027E-13 | 130 |
| **hsa05415** | Diabetic cardiomyopathy | 170/4907 | 203/8114 | 2.96748904001303E-13 | 6.56804240856217E-12 | 3.47768891005035E-12 | 170 |
| **hsa04218** | Cellular senescence | 135/4907 | 156/8114 | 5.12576229174699E-13 | 1.0635956755375E-11 | 5.6315940968536E-12 | 135 |
| **hsa05210** | Colorectal cancer | 81/4907 | 86/8114 | 6.05179608859069E-13 | 1.18188017730124E-11 | 6.25789440739719E-12 | 81 |
| **hsa05168** | Herpes simplex virus 1 infection | 372/4907 | 495/8114 | 9.49718342741326E-13 | 1.75170272105622E-11 | 9.27502708992991E-12 | 372 |
| **hsa05022** | Pathways of neurodegeneration - multiple diseases | 359/4907 | 476/8114 | 1.05250575722169E-12 | 1.83911532314527E-11 | 9.73786490061065E-12 | 359 |
| **hsa03013** | Nucleocytoplasmic transport | 98/4907 | 108/8114 | 1.26679243472595E-12 | 2.10287544164508E-11 | 1.11344387683807E-11 | 98 |
| **hsa05131** | Shigellosis | 200/4907 | 247/8114 | 1.87548819419266E-12 | 2.96505752605697E-11 | 1.56995753599085E-11 | 200 |
| **hsa04110** | Cell cycle | 111/4907 | 126/8114 | 4.80239961192997E-12 | 7.24725759618522E-11 | 3.83732409182921E-11 | 111 |
| **hsa05220** | Chronic myeloid leukemia | 72/4907 | 76/8114 | 5.38269359685404E-12 | 7.76980119198061E-11 | 4.11400380171453E-11 | 72 |
| **hsa05020** | Prion disease | 217/4907 | 273/8114 | 7.34240727363435E-12 | 1.01569967285275E-10 | 5.37799129253043E-11 | 217 |
| **hsa05212** | Pancreatic cancer | 71/4907 | 76/8114 | 5.25698107264092E-11 | 6.98127086446715E-10 | 3.69648774370962E-10 | 71 |
| **hsa04142** | Lysosome | 113/4907 | 132/8114 | 1.8798766029176E-10 | 2.40045781603324E-09 | 1.27100968699287E-09 | 113 |
| **hsa03010** | Ribosome | 132/4907 | 158/8114 | 1.99716924212896E-10 | 2.45577847550672E-09 | 1.3003012219709E-09 | 132 |
| **hsa04137** | Mitophagy - animal | 67/4907 | 72/8114 | 3.05628970995627E-10 | 3.62388637037672E-09 | 1.91879842692743E-09 | 67 |
| **hsa03018** | RNA degradation | 72/4907 | 79/8114 | 8.23200712592631E-10 | 9.42422884761219E-09 | 4.99000068976295E-09 | 72 |
| **hsa04150** | mTOR signaling pathway | 129/4907 | 156/8114 | 1.27063578727007E-09 | 1.40617027124554E-08 | 7.44547987628426E-09 | 129 |
| **hsa05161** | Hepatitis B | 132/4907 | 162/8114 | 5.48331834189324E-09 | 5.87245706293082E-08 | 3.10938595278836E-08 | 132 |
| **hsa05166** | Human T-cell leukemia virus 1 infection | 174/4907 | 222/8114 | 6.92095484382333E-09 | 7.1804906504667E-08 | 3.80197190433716E-08 | 174 |
| **hsa05211** | Renal cell carcinoma | 63/4907 | 69/8114 | 8.2084975661359E-09 | 8.2582460362337E-08 | 4.37262868754289E-08 | 63 |
| **hsa04068** | FoxO signaling pathway | 109/4907 | 131/8114 | 1.24340340320029E-08 | 1.21414685253675E-07 | 6.42874205369809E-08 | 109 |
| **hsa04211** | Longevity regulating pathway | 78/4907 | 89/8114 | 1.33868134591828E-08 | 1.26983487669962E-07 | 6.72360254942413E-08 | 78 |
| **hsa04910** | Insulin signaling pathway | 113/4907 | 137/8114 | 1.83238058636651E-08 | 1.68986209631578E-07 | 8.94758941295929E-08 | 113 |
| **hsa01524** | Platinum drug resistance | 65/4907 | 73/8114 | 5.54271099065458E-08 | 4.97345959161438E-07 | 2.63337904819151E-07 | 65 |
| **hsa04722** | Neurotrophin signaling pathway | 99/4907 | 119/8114 | 5.95574363180455E-08 | 5.20343917305029E-07 | 2.7551501011395E-07 | 99 |
| **hsa05203** | Viral carcinogenesis | 159/4907 | 204/8114 | 6.26348687564443E-08 | 5.3319939556768E-07 | 2.82321810589101E-07 | 159 |
| **hsa04520** | Adherens junction | 63/4907 | 71/8114 | 1.21759689651614E-07 | 1.0106054241084E-06 | 5.35101793995251E-07 | 63 |
| **hsa05213** | Endometrial cancer | 53/4907 | 58/8114 | 1.25165663846677E-07 | 1.01353659505114E-06 | 5.36653809047371E-07 | 53 |
| **hsa04668** | TNF signaling pathway | 93/4907 | 112/8114 | 1.81060540547809E-07 | 1.43124046337792E-06 | 7.57822312568522E-07 | 93 |
| **hsa03015** | mRNA surveillance pathway | 82/4907 | 97/8114 | 2.02644634039829E-07 | 1.5646050814238E-06 | 8.28437059599792E-07 | 82 |
| **hsa04210** | Apoptosis | 110/4907 | 136/8114 | 2.22918519934449E-07 | 1.68202155950539E-06 | 8.90607483948637E-07 | 110 |
| **hsa04115** | p53 signaling pathway | 64/4907 | 73/8114 | 2.72749092162164E-07 | 2.01228219106307E-06 | 1.06547598575629E-06 | 64 |
| **hsa03050** | Proteasome | 43/4907 | 46/8114 | 3.98456721376574E-07 | 2.87581807602223E-06 | 1.52270646384183E-06 | 43 |
| **hsa04152** | AMPK signaling pathway | 98/4907 | 120/8114 | 4.2391506477926E-07 | 2.99446386184499E-06 | 1.58552778987988E-06 | 98 |
| **hsa01521** | EGFR tyrosine kinase inhibitor resistance | 68/4907 | 79/8114 | 5.49822796140872E-07 | 3.80294100664103E-06 | 2.01360541569135E-06 | 68 |
| **hsa05225** | Hepatocellular carcinoma | 131/4907 | 168/8114 | 8.76653013723456E-07 | 5.93977143992219E-06 | 3.14502799767599E-06 | 131 |
| **hsa05100** | Bacterial invasion of epithelial cells | 66/4907 | 77/8114 | 1.13057848914283E-06 | 7.50704116790841E-06 | 3.97487595130217E-06 | 66 |
| **hsa00280** | Valine, leucine and isoleucine degradation | 44/4907 | 48/8114 | 1.26532960793247E-06 | 8.1087604785472E-06 | 4.29347812275645E-06 | 44 |
| **hsa05205** | Proteoglycans in cancer | 156/4907 | 205/8114 | 1.27004682194113E-06 | 8.1087604785472E-06 | 4.29347812275645E-06 | 156 |
| **hsa05017** | Spinocerebellar ataxia | 113/4907 | 143/8114 | 1.49266153671142E-06 | 9.35025717336211E-06 | 4.95083369673897E-06 | 113 |
| **hsa04136** | Autophagy - other | 31/4907 | 32/8114 | 2.17034174639817E-06 | 1.33435825889666E-05 | 7.06524506137418E-06 | 31 |
| **hsa05215** | Prostate cancer | 80/4907 | 97/8114 | 2.29586838690991E-06 | 1.38586964446198E-05 | 7.33799082514748E-06 | 80 |
| **hsa05135** | Yersinia infection | 108/4907 | 137/8114 | 3.14582934648093E-06 | 1.86502739827083E-05 | 9.87506580568261E-06 | 108 |
| **hsa05222** | Small cell lung cancer | 76/4907 | 92/8114 | 3.64414785006436E-06 | 2.12255629161643E-05 | 1.12386461857941E-05 | 76 |
| **hsa03030** | DNA replication | 34/4907 | 36/8114 | 3.87549636155537E-06 | 2.21838757247652E-05 | 1.17460597528085E-05 | 34 |
| **hsa05163** | Human cytomegalovirus infection | 168/4907 | 225/8114 | 4.00356044300855E-06 | 2.25285096115058E-05 | 1.19285386972779E-05 | 168 |
| **hsa04070** | Phosphatidylinositol signaling system | 79/4907 | 97/8114 | 6.96152747508787E-06 | 3.85204520288195E-05 | 2.03960541813978E-05 | 79 |
| **hsa04933** | AGE-RAGE signaling pathway in diabetic complications | 81/4907 | 100/8114 | 8.0332283350582E-06 | 4.37218329055627E-05 | 2.31501144427044E-05 | 81 |
| **hsa05170** | Human immunodeficiency virus 1 infection | 158/4907 | 212/8114 | 9.28733243792858E-06 | 4.97321672482627E-05 | 2.63325045353832E-05 | 158 |
| **hsa03020** | RNA polymerase | 32/4907 | 34/8114 | 9.53113701611922E-06 | 5.02275791960569E-05 | 2.65948184075507E-05 | 32 |
| **hsa03420** | Nucleotide excision repair | 42/4907 | 47/8114 | 1.14082628698693E-05 | 5.9180363637447E-05 | 3.13351957116476E-05 | 42 |
| **hsa04071** | Sphingolipid signaling pathway | 94/4907 | 119/8114 | 1.17166523595544E-05 | 5.9845055128801E-05 | 3.16871407942605E-05 | 94 |
| **hsa05120** | Epithelial cell signaling in Helicobacter pylori infection | 59/4907 | 70/8114 | 1.31680645535484E-05 | 6.62393550269406E-05 | 3.50728354137574E-05 | 59 |
| **hsa04510** | Focal adhesion | 150/4907 | 201/8114 | 1.3791686840358E-05 | 6.83408959850574E-05 | 3.61855726997609E-05 | 150 |
| **hsa05231** | Choline metabolism in cancer | 79/4907 | 98/8114 | 1.4678183264061E-05 | 7.16640712304154E-05 | 3.79451486857304E-05 | 79 |
| **hsa03410** | Base excision repair | 31/4907 | 33/8114 | 1.490962423996E-05 | 7.17390615603875E-05 | 3.79848550430714E-05 | 31 |
| **hsa04213** | Longevity regulating pathway - multiple species | 53/4907 | 62/8114 | 1.57737711185444E-05 | 7.48127430193822E-05 | 3.96123274706304E-05 | 53 |
| **hsa05167** | Kaposi sarcoma-associated herpesvirus infection | 145/4907 | 194/8114 | 1.66454804827527E-05 | 7.75120859776666E-05 | 4.10415927656003E-05 | 145 |
| **hsa04919** | Thyroid hormone signaling pathway | 95/4907 | 121/8114 | 1.68098499710602E-05 | 7.75120859776666E-05 | 4.10415927656003E-05 | 95 |
| **hsa05165** | Human papillomavirus infection | 236/4907 | 331/8114 | 1.80798602457614E-05 | 8.22262137204489E-05 | 4.35376591354311E-05 | 236 |
| **hsa01212** | Fatty acid metabolism | 49/4907 | 57/8114 | 2.41602252538468E-05 | 1.08394524111853E-04 | 5.73934227225096E-05 | 49 |
| **hsa05418** | Fluid shear stress and atherosclerosis | 107/4907 | 139/8114 | 2.47714316671101E-05 | 1.09654870846407E-04 | 5.80607591355422E-05 | 107 |
| **hsa00520** | Amino sugar and nucleotide sugar metabolism | 43/4907 | 49/8114 | 2.57992843455741E-05 | 1.12702136878034E-04 | 5.96742449544442E-05 | 43 |
| **hsa04012** | ErbB signaling pathway | 69/4907 | 85/8114 | 3.29749198247262E-05 | 1.42177576387131E-04 | 7.52810883216579E-05 | 69 |
| **hsa04931** | Insulin resistance | 85/4907 | 108/8114 | 3.94709597924451E-05 | 1.6800459809092E-04 | 8.89561442015969E-05 | 85 |
| **hsa00310** | Lysine degradation | 53/4907 | 63/8114 | 4.09056596792678E-05 | 1.71907329285024E-04 | 9.10225871610622E-05 | 53 |
| **hsa05169** | Epstein-Barr virus infection | 149/4907 | 202/8114 | 4.19803863822078E-05 | 1.74218603486162E-04 | 9.22463753398513E-05 | 149 |
| **hsa04010** | MAPK signaling pathway | 210/4907 | 294/8114 | 4.33953421968837E-05 | 1.77867328510684E-04 | 9.41783254955112E-05 | 210 |
| **hsa00562** | Inositol phosphate metabolism | 60/4907 | 73/8114 | 5.30598782515874E-05 | 2.1482779975033E-04 | 1.1374839111701E-04 | 60 |
| **hsa04360** | Axon guidance | 135/4907 | 182/8114 | 6.09216684043646E-05 | 2.43686673617458E-04 | 1.29028771382738E-04 | 135 |
| **hsa00510** | N-Glycan biosynthesis | 43/4907 | 50/8114 | 7.62343185997819E-05 | 3.01307068751519E-04 | 1.59537985039644E-04 | 43 |
| **hsa01200** | Carbon metabolism | 89/4907 | 115/8114 | 8.32198943749455E-05 | 3.25047116852728E-04 | 1.72108016849732E-04 | 89 |
| **hsa03022** | Basal transcription factors | 39/4907 | 45/8114 | 1.16216054808606E-04 | 4.46573011212762E-04 | 2.36454321092363E-04 | 39 |
| **hsa01250** | Biosynthesis of nucleotide sugars | 33/4907 | 37/8114 | 1.17023650528645E-04 | 4.46573011212762E-04 | 2.36454321092363E-04 | 33 |
| **hsa03060** | Protein export | 22/4907 | 23/8114 | 1.49384096778671E-04 | 5.63585456028624E-04 | 2.98410815335384E-04 | 22 |
| **hsa04370** | VEGF signaling pathway | 49/4907 | 59/8114 | 1.56451763964732E-04 | 5.836178161381E-04 | 3.09017676902545E-04 | 49 |
| **hsa05223** | Non-small cell lung cancer | 58/4907 | 72/8114 | 2.11288167202593E-04 | 7.79418572347345E-04 | 4.12691507869393E-04 | 58 |
| **hsa05214** | Glioma | 60/4907 | 75/8114 | 2.37305936870585E-04 | 8.65775505945432E-04 | 4.58416326863941E-04 | 60 |
| **hsa01522** | Endocrine resistance | 76/4907 | 98/8114 | 2.42031325244566E-04 | 8.72967908226369E-04 | 4.62224605814216E-04 | 76 |
| **hsa04710** | Circadian rhythm | 28/4907 | 31/8114 | 2.44536191159796E-04 | 8.72967908226369E-04 | 4.62224605814216E-04 | 28 |
| **hsa05110** | Vibrio cholerae infection | 42/4907 | 50/8114 | 2.83572588915129E-04 | 0.001001554 | 5.30309320815527E-04 | 42 |
| **hsa04350** | TGF-beta signaling pathway | 73/4907 | 94/8114 | 2.9615790371314E-04 | 0.001034994 | 5.48015179170021E-04 | 73 |
| **hsa00020** | Citrate cycle (TCA cycle) | 27/4907 | 30/8114 | 3.68033103567547E-04 | 0.001272781 | 6.739202664011E-04 | 27 |
| **hsa05171** | Coronavirus disease - COVID-19 | 165/4907 | 232/8114 | 3.9636571740178E-04 | 0.001356633 | 7.18318771634263E-04 | 165 |
| **hsa05216** | Thyroid cancer | 32/4907 | 37/8114 | 5.38380855135955E-04 | 0.001823902 | 9.65731501693926E-04 | 32 |
| **hsa04130** | SNARE interactions in vesicular transport | 29/4907 | 33/8114 | 5.60653890626803E-04 | 0.001880173 | 9.95525781336269E-04 | 29 |
| **hsa00532** | Glycosaminoglycan biosynthesis - chondroitin sulfate / dermatan sulfate | 19/4907 | 20/8114 | 5.95462796501586E-04 | 0.001976936 | 0.001046761 | 19 |
| **hsa01240** | Biosynthesis of cofactors | 112/4907 | 153/8114 | 6.02002593938447E-04 | 0.00197886 | 0.001047779 | 112 |
| **hsa00240** | Pyrimidine metabolism | 47/4907 | 58/8114 | 6.56084133976631E-04 | 0.00213549 | 0.001130713 | 47 |
| **hsa04935** | Growth hormone synthesis, secretion and action | 89/4907 | 119/8114 | 6.76489955547614E-04 | 0.002180531 | 0.001154561 | 89 |
| **hsa03460** | Fanconi anemia pathway | 44/4907 | 54/8114 | 7.79071153451575E-04 | 0.002487035 | 0.001316851 | 44 |
| **hsa00640** | Propanoate metabolism | 28/4907 | 32/8114 | 8.22372789855964E-04 | 0.002600264 | 0.001376805 | 28 |
| **hsa05221** | Acute myeloid leukemia | 53/4907 | 67/8114 | 9.08692298552876E-04 | 0.002846093 | 0.001506967 | 53 |
| **hsa05235** | PD-L1 expression and PD-1 checkpoint pathway in cancer | 68/4907 | 89/8114 | 0.001068691 | 0.003315939 | 0.001755744 | 68 |
| **hsa03430** | Mismatch repair | 21/4907 | 23/8114 | 0.001160631 | 0.003567866 | 0.001889136 | 21 |
| **hsa04146** | Peroxisome | 63/4907 | 82/8114 | 0.001259981 | 0.00383774 | 0.002032031 | 63 |
| **hsa00563** | Glycosylphosphatidylinositol (GPI)-anchor biosynthesis | 23/4907 | 26/8114 | 0.001824339 | 0.005506187 | 0.002915451 | 23 |
| **hsa04962** | Vasopressin-regulated water reabsorption | 36/4907 | 44/8114 | 0.002051604 | 0.006086447 | 0.003222691 | 36 |
| **hsa04926** | Relaxin signaling pathway | 94/4907 | 129/8114 | 0.002053259 | 0.006086447 | 0.003222691 | 94 |
| **hsa05230** | Central carbon metabolism in cancer | 54/4907 | 70/8114 | 0.002354659 | 0.006918112 | 0.003663046 | 54 |
| **hsa04392** | Hippo signaling pathway - multiple species | 25/4907 | 29/8114 | 0.002533698 | 0.007378839 | 0.003906994 | 25 |
| **hsa04936** | Alcoholic liver disease | 102/4907 | 142/8114 | 0.002932079 | 0.008464785 | 0.004481988 | 102 |
| **hsa05130** | Pathogenic Escherichia coli infection | 138/4907 | 197/8114 | 0.002988375 | 0.008552935 | 0.004528663 | 138 |
| **hsa04666** | Fc gamma R-mediated phagocytosis | 72/4907 | 97/8114 | 0.003021985 | 0.008575205 | 0.004540454 | 72 |
| **hsa04215** | Apoptosis - multiple species | 27/4907 | 32/8114 | 0.003249665 | 0.009143126 | 0.004841161 | 27 |
| **hsa01230** | Biosynthesis of amino acids | 57/4907 | 75/8114 | 0.003295974 | 0.009195492 | 0.004868888 | 57 |
| **hsa00513** | Various types of N-glycan biosynthesis | 32/4907 | 39/8114 | 0.003346145 | 0.009257668 | 0.004901809 | 32 |
| **hsa04810** | Regulation of actin cytoskeleton | 151/4907 | 218/8114 | 0.003963057 | 0.010873842 | 0.005757551 | 151 |
| **hsa04917** | Prolactin signaling pathway | 53/4907 | 70/8114 | 0.005210148 | 0.013709687 | 0.007259092 | 53 |
| **hsa03440** | Homologous recombination | 33/4907 | 41/8114 | 0.00521529 | 0.013709687 | 0.007259092 | 33 |
| **hsa04216** | Ferroptosis | 33/4907 | 41/8114 | 0.00521529 | 0.013709687 | 0.007259092 | 33 |
| **hsa05219** | Bladder cancer | 33/4907 | 41/8114 | 0.00521529 | 0.013709687 | 0.007259092 | 33 |
| **hsa00062** | Fatty acid elongation | 23/4907 | 27/8114 | 0.005244368 | 0.013709687 | 0.007259092 | 23 |
| **hsa01040** | Biosynthesis of unsaturated fatty acids | 23/4907 | 27/8114 | 0.005244368 | 0.013709687 | 0.007259092 | 23 |
| **hsa04066** | HIF-1 signaling pathway | 79/4907 | 109/8114 | 0.005722194 | 0.014841939 | 0.007858605 | 79 |
| **hsa00920** | Sulfur metabolism | 10/4907 | 10/8114 | 0.006519807 | 0.016779659 | 0.008884601 | 10 |
| **hsa04914** | Progesterone-mediated oocyte maturation | 74/4907 | 102/8114 | 0.007073772 | 0.018065326 | 0.009565344 | 74 |
| **hsa04912** | GnRH signaling pathway | 68/4907 | 93/8114 | 0.007150026 | 0.018120677 | 0.009594652 | 68 |
| **hsa04390** | Hippo signaling pathway | 110/4907 | 157/8114 | 0.007490955 | 0.018840887 | 0.009975993 | 110 |
| **hsa04922** | Glucagon signaling pathway | 77/4907 | 107/8114 | 0.008466808 | 0.021135191 | 0.011190795 | 77 |
| **hsa04625** | C-type lectin receptor signaling pathway | 75/4907 | 104/8114 | 0.008553521 | 0.021192306 | 0.011221037 | 75 |
| **hsa00511** | Other glycan degradation | 16/4907 | 18/8114 | 0.009086506 | 0.022346073 | 0.011831941 | 16 |
| **hsa05417** | Lipid and atherosclerosis | 147/4907 | 215/8114 | 0.009259825 | 0.022604867 | 0.011968969 | 147 |
| **hsa04621** | NOD-like receptor signaling pathway | 127/4907 | 184/8114 | 0.009382546 | 0.022737265 | 0.012039072 | 127 |
| **hsa05160** | Hepatitis C | 109/4907 | 157/8114 | 0.011846338 | 0.028499887 | 0.015090301 | 109 |
| **hsa04072** | Phospholipase D signaling pathway | 103/4907 | 148/8114 | 0.012766838 | 0.030493455 | 0.016145869 | 103 |
| **hsa05218** | Melanoma | 53/4907 | 72/8114 | 0.013484622 | 0.031824402 | 0.016850587 | 53 |
| **hsa00450** | Selenocompound metabolism | 15/4907 | 17/8114 | 0.013515785 | 0.031824402 | 0.016850587 | 15 |
| **hsa03450** | Non-homologous end-joining | 12/4907 | 13/8114 | 0.013687502 | 0.032001765 | 0.016944498 | 12 |
| **hsa00230** | Purine metabolism | 89/4907 | 127/8114 | 0.015077264 | 0.035004557 | 0.018534436 | 89 |
| **hsa00270** | Cysteine and methionine metabolism | 38/4907 | 50/8114 | 0.015456355 | 0.035635486 | 0.018868504 | 38 |
| **hsa04340** | Hedgehog signaling pathway | 42/4907 | 56/8114 | 0.016179117 | 0.0370446 | 0.019614611 | 42 |
| **hsa04114** | Oocyte meiosis | 91/4907 | 131/8114 | 0.019900403 | 0.04525297 | 0.023960831 | 91 |
| **hsa00051** | Fructose and mannose metabolism | 26/4907 | 33/8114 | 0.020956358 | 0.047330007 | 0.025060593 | 26 |
| **hsa05162** | Measles | 96/4907 | 139/8114 | 0.021488231 | 0.048203328 | 0.025523005 | 96 |
